# *BRCA1* and *BRCA2* pathogenic variants and prostate cancer risk: systematic review and meta-analysis

**DOI:** 10.1038/s41416-021-01675-5

**Published:** 2021-12-28

**Authors:** Tommy Nyberg, Marc Tischkowitz, Antonis C. Antoniou

**Affiliations:** 1 Centre for Cancer Genetic Epidemiology, Department of Public Health and Primary Care, University of Cambridge, Cambridge, UK; 2 MRC Biostatistics Unit, University of Cambridge, Cambridge, UK; 3 Department of Medical Genetics, National Institute for Health Research Cambridge Biomedical Research Centre, University of Cambridge, Cambridge, UK

**Keywords:** Cancer epidemiology, Cancer epigenetics, Prostate cancer, Risk factors

## Abstract

**Background:**

*BRCA1* and *BRCA2* pathogenic variants (PVs) are associated with prostate cancer (PCa) risk, but a wide range of relative risks (RRs) has been reported.

**Methods:**

We systematically searched PubMed, Embase, MEDLINE and Cochrane Library in June 2021 for studies that estimated PCa RRs for male *BRCA1/2* carriers, with no time or language restrictions. The literature search identified 27 studies (*BRCA1*: *n* = 20, *BRCA2*: *n* = 21).

**Results:**

The heterogeneity between the published estimates was high (*BRCA1*: *I*^2^ = 30%, *BRCA2*: *I*^2^ = 83%); this could partly be explained by selection for age, family history or aggressive disease, and study-level differences in ethnicity composition, use of historical controls, and location of PVs within *BRCA2*. The pooled RRs were 2.08 (95% CI 1.38–3.12) for Ashkenazi Jewish *BRCA2* carriers, 4.35 (95% CI 3.50–5.41) for non-Ashkenazi European ancestry *BRCA2* carriers, and 1.18 (95% CI 0.95–1.47) for *BRCA1* carriers. At ages <65 years, the RRs were 7.14 (95% CI 5.33–9.56) for non-Ashkenazi European ancestry *BRCA2* and 1.78 (95% CI 1.09–2.91) for *BRCA1* carriers.

**Conclusions:**

These PCa risk estimates will assist in guiding clinical management. The study-level subgroup analyses indicate that risks may be modified by age and ethnicity, and for *BRCA2* carriers by PV location within the gene, which may guide future risk-estimation studies.

## Introduction

Pathogenic variants (PVs) in *BRCA1* and *BRCA2* are associated with prostate cancer (PCa) risk, but a wide range of relative risk (RR) estimates has been reported [1–26]. A systematic review and meta-analysis on PCa risks for men with germline *BRCA1/2* PVs (henceforth, “*BRCA1/2* carriers”) was published in 2019, and estimated pooled RRs of 1.35 (95% CI 1.03–1.76) for *BRCA1* and 2.64 (95% CI 2.03–3.47) for *BRCA2* carriers [27]. However, that meta-analysis did not consider variation in the RRs by age, PCa family history, ethnicity or PV location despite evidence of variation by these factors [1–8, 10–12, 14–17, 23, 28–33], and did not include two subsequent studies that reported prospective RR estimates for *BRCA1/2* carriers: the IMPACT screening trial [20] and the EMBRACE study [23].

### Study aims

This systematic review and meta-analysis aimed to synthesise the available evidence on the RRs of PCa for male *BRCA1* and *BRCA2* carriers, overall and by age groups, and to explore potential explanatory factors for the variation in the reported estimates by study-level covariates. Secondarily, we aimed to estimate RRs of PCa applicable to *BRCA1/2* carriers with a PCa family history, and RRs of aggressive PCa.

## Methods

We sought to identify all available estimates of the RRs of PCa for *BRCA1/2* carriers, based on valid study designs [34]. On June 19, 2021, the first author (TN) searched PubMed, Embase, MEDLINE and Cochrane Library with no time or language restrictions. The search query is available in the Supplementary Material. The first author removed duplicates, conference abstracts and publications that did not report original data, and screened the remaining publications based on their titles and abstracts to identify those potentially relevant. The first author thereafter screened these articles in their entirety. We contacted the authors of five articles to ask for clarifications.

We included case–control, prospective cohort and family-based retrospective cohort studies [34] that estimated the RR and 95% CI of diagnosed PCa (regardless of histopathology) for carriers of rare PVs in *BRCA1* and/or *BRCA2* compared to the general population or to non-carriers, or studies where RRs and/or CIs were not reported but the study provided sufficient information to allow calculation of the missing measures. Whenever available, we used estimates adjusted for age and/or ancestry as reported in the publications. PVs were defined as any deleterious variants as determined by the study investigators or in a clinical setting to be clinically actionable based on established clinical guidelines. Studies that only reported on PVs in the two genes together, without providing separate risk estimates for *BRCA1* and *BRCA2* PVs, were not included. We did not include retrospective cohort studies that recruited PV carriers in clinical settings and assessed association with previous cancer diagnoses, because of the likely ascertainment bias associated with such study designs; [34] nor cross-sectional studies that compared frequencies of prevalent PCa between PV carriers and non-carriers, because prevalence ratios are unbiased RR estimates only under strong assumptions about the population incidence [35]. When data from the same study had been published more than once, we only included the most recent publication.

### Statistical analysis

We used the DerSimonian—Laird method for the between-study variance [36] and derived pooled estimates according to both fixed-effects and random-effects models. To assess heterogeneity between RR estimates, we used the DerSimonian—Laird heterogeneity of effects chi-square test and reported the corresponding *I*^2^ statistic [37]. We assessed whether the study estimates varied by covariate moderators using nested chi-square tests for categorical moderators or meta-regression for quantitative moderators [38]. To assess potential publication bias, we used funnel plots and tested for funnel plot asymmetry using the rank correlation test [39]. To assess the impact of individual studies on the results, we performed leave-one-out sensitivity analyses by omitting one of the included studies at a time and refitting the models.

For the meta-analysis by age groups, we initially considered all reported estimates by age at diagnosis with no restriction on age cutpoints considered, and also specifically those that used an age cutpoint of 65 years. For the meta-analysis of aggressive PCa, we considered studies that had exclusively or preferentially included participants with aggressive PCa, or studies that reported aggressive PCa-specific RRs, with PCa aggressiveness as defined by the study authors. In addition, when no RR of aggressive PCa had been reported but sufficient data were available within a study (e.g. Gleason score frequencies for PCa cases by PV status), we estimated the RR of Gleason score ≥7 PCa. We explored whether the variability between the estimates could be explained by the following study-level covariates (defined in Supplementary Table S1): study design; the majority ethnic ancestry of the study participants; age-adjustment approach; participant, case or control selection; use of historical or external controls; and the proportion of observed *BRCA2* PVs that were located within the wide definition ovarian cancer cluster region (OCCR) [8, 16, 23, 29, 30, 32, 40]. We performed the meta-analyses using R software [41], with the *meta* [42] and *metafor* [38] packages.

## Results

**Fig. 1:**
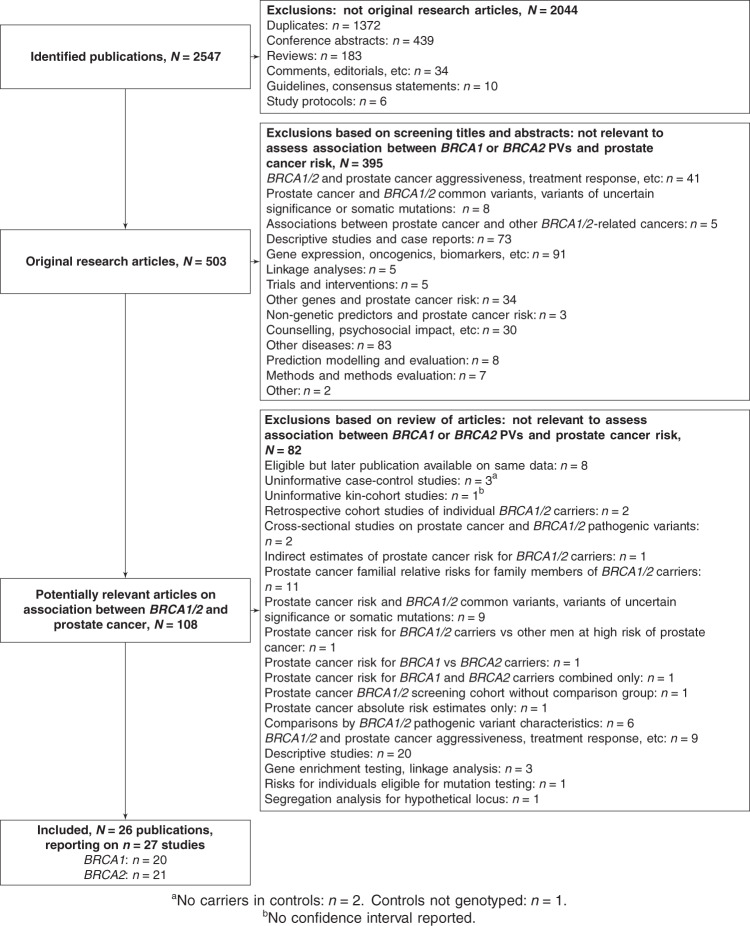
Flowchart. Flowchart detailing the identification of original research articles on the relative risk of prostate cancer for carriers of *BRCA1* and *BRCA2* pathogenic variants.

The literature search identified 27 studies that reported PCa RR estimates for *BRCA1* (*n* = 20) and/or *BRCA2* carriers (*n* = 21; Fig. 1). These included 20 case–control studies from 19 publications [1, 3, 4, 6, 7, 10–15, 17–19, 21, 22, 24–26], two prospective cohort studies [20, 23], and five family-based retrospective cohort studies [2, 5, 8, 9, 16] (Tables 1 and 2). Full details are available in the Supplementary Material.

**Table 1:** Case–control studies.

Publication	Population, dataset	Period	Study design	Selection	Cases average age	Controls average age	Age-adjustment	Gene	Considered PVs	% PVs located in *BRCA2* OCCR	Cases PV carriers/total (%)	Controls PV carriers/total (%)	OR (95% CI)^a^
Johannesdottir [1]	Iceland	1983–1992	Cases vs controls from the same population	Cases: men diagnosed with PCa at age <65 at a single clinic (University Hospital of Iceland). Controls: participants in an unrelated public health study.	Not stated (all <65)	Not stated	None	*BRCA2*	c.771_775del	0% by design	2/75 (2.67%)	2/499 (0.40%)	6.6 (0.81–56.9)
Hubert [3]	Israel	Not stated	Cases vs controls from the same population	Cases: unselected men diagnosed with PCa at a single clinic (Sharett Institute, Hadassah Hebrew University Hospital). Controls: recruited from homes for the elderly.	Median: 71	Median: 72	None, but cases and controls were of comparable ages	*BRCA1*	c.68_69delAG		2/87 (2.30%)	2/87 (2.30%)	Not reported
								*BRCA2*	c.5946delT	100% by design	1/87 (1.15%)	1/87 (1.15%)	Not reported
Vazina [4]	Israel	1998	Cases vs historical controls	Cases: unselected men diagnosed with PCa at three clinics (Rabin, Sheba or the Wolfson Medical Centers). Controls: historical US Ashkenazi controls [50].	Median: 66	Not stated (historical controls)	None	*BRCA1*	c.68_69delAG		4/87 (4.60%)	61/5318 (1.15%)	Not reported
								*BRCA2*	c.5946delT	100% by design	1/86 (1.16%)	59/5087(1.16%)	Not reported
Giusti [6]	Israel	1994–1995	Cases vs historical controls	Cases: unselected men diagnosed with PCa at 16 clinics. Controls: historical controls from the US Ashkenazi population [50] and an Israeli colorectal cancer case–control study [51].	Mean: 73.6	Not stated (historical controls)	None	*BRCA1*	c.68_69delAG and c.5266dupC		16/940 (1.70%)	11/1344 (0.82%)	Not reported
								*BRCA2*	c.5946delT	100% by design	14/940 (1.49%)	10/1344 (0.74%)	2.02 (0.89–4.56)
Hamel [7]^b^	Canadian Ashkenazi	1991–2002	Cases vs historical controls	Cases: unselected Ashkenazi men diagnosed with PCa at three clinics in Montreal. Controls: historical controls from five studies with Ashkenazi general population or study control groups.	Mean: 67.9	Not stated (historical controls)	None	*BRCA1*	c.68_69delAG and c.5266dupC		0/146 (0.00%)	109/9371 (1.16%)	Not reported
								*BRCA2*	c.5946delT	100% by design	2/146 (1.37%)	119/9514 (1.25%)	Not reported
Agalliu[10]	USA (predominantly European ancestry)	1993–1996, 2002–2005	Cases vs population frequency estimate	Cases: men diagnosed with PCa at age <55 in two case–control studies. No controls; comparison to a previous population *BRCA2* frequency estimate for US Caucasians.	Median: 49.5	--	None	*BRCA2*	c.3847_3848del and c.4398_4402del	2/2 (100%)	2/257 (0.78%)	Population frequency: 0.1%	7.78 (1.80–9.37)
Agalliu [11]	US Ashkenazi	1998–2005	Cases vs controls from the same population	Cases and controls: self-selected Ashkenazi volunteers who were recruited through advertisements. The participants provided self-reported case/control status.	Mean: 69.4	Mean: 68.3	Covariate adjustment for age	*BRCA1*	c.68_69delAG and c.5266dupC		12/978 (1.23%)	11/1247 (0.88%)	1.39 (0.60–3.22)
								*BRCA2*	c.5946delT	100% by design	18/969 (1.86%)	12/1240 (0.97%)	1.92 (0.91–4.07)
Gallagher [12]	US Ashkenazi, MSKCC	1988–2007	Cases vs controls from the same population	Cases: unselected Ashkenazi men treated with PCa at a single clinic (Memorial Sloan-Kettering Cancer Center, New York). Controls: Ashkenazi healthy volunteers from a prospective study in New York.	Median: 68	Median: 42	Covariate adjustment for age	*BRCA1*	c.68_69delAG		6/832 (0.72%)	4/454 (0.88%)	0.38 (0.05–2.75)
								*BRCA2*	c.5946delT	100% by design	20/832 (2.40%)	3/454 (0.66%)	3.18 (1.52–6.66)
Fachal [13]	Spain	2006–2009	Cases vs controls from the same population	Cases: unselected men treated for PCa at one clinic (Santiago de Compostela). Controls: healthy men aged >44 (selection unclear).	Median: 68	Median: 60	Covariate adjustment for age	*BRCA1*	c.211 A > G		1/905 (0.11%)	3/936 (0.32%)	0.27 (0.01–2.36)
Kote-Jarai [14]	UK, UKGPCS	Not stated	Cases vs population frequency estimate	Cases: men with PCa recruited nationwide due to being diagnosed with PCa at age <65 years (87% of the study sample), or due to having a family history of PCa (13% of the study sample). No controls; comparison to a previous UK population *BRCA2* frequency estimate.	Not stated (87% <65)	--	None	*BRCA2*	Any pathogenic variant	11/19 (58%)	19/1832 (1.04%)	Population frequency: 0.16%	8.6 (5.1–12.6)
Leongamornlert [15]	UK, UKGPCS	Not stated	Cases vs population frequency estimate	Cases: men with PCa recruited nationwide due to being diagnosed with PCa at age <65 years (90% of the study sample), or due to having a family history of PCa (10% of the study sample). No controls; comparison to a previous UK population *BRCA1* frequency estimate.	Not stated (90% <65)	--	None	*BRCA1*	Any pathogenic variant		4/886 (0.45%)	Population frequency: 0.12%	3.75 (1.02–9.60)
Cybulski [17]	Poland	1999–2012	Cases vs controls from the same population	Cases: unselected men with PCa from 14 centres. Controls: population controls from four sources (a random clinic record sample, a population-based study, PSA-screen negative men, colonoscopy screening participants).	Mean: 68.8	Mean: 61.2	None	*BRCA1*	c.181 T > G, c.4035del and c.5266dupC		14/3750 (0.37%)	17/3956 (0.43%)	0.9 (0.4–1.8)
Akbari [18]	Canada (predominantly European ancestry)	1998–2010	Cases vs controls from the same population	Cases and controls: unselected men who had a biopsy because of elevated PSA or abnormal DRE at two clinics; cases were those biopsy-positive, controls were those biopsy-negative.	Mean: 65	Not stated	None, but cases and controls were likely of comparable ages	*BRCA2*	Any pathogenic variant	Not specified	26/1904 (1.37%)	9/2283 (0.39%)	3.5 (1.63–7.48)
Pritchard [19]	UK and USA (predominantly European ancestry)	Not stated	Cases vs population frequency estimate	Cases: men with metastatic PCa from seven case series. No controls; comparison to carrier frequency in the Exome Aggregation Consortium database.	Not stated	Not stated (external estimate)	None	*BRCA1*	Any pathogenic variant		6/692 (0.87%)	104/53105 (0.20%)	3.9 (1.4–8.5)
								*BRCA2*	Any pathogenic variant	24/37 (65%)	37/692 (5.35%)	153/53105 (0.29%)	18.6 (13.2–25.3)
Matejcic [21]	US African Americans (AA)	1993–2015	Cases vs controls from the same population	Cases: men with PCa “overselected for high stage and Gleason score” from incident cases from a US prospective cohort study and two US case series of African American participants. Controls: unaffected African American participants in the US prospective cohort study.	Mean: 66.71	Mean: 71.52	Covariate adjustment for age and genetic ancestry	*BRCA1*	Any pathogenic variant		3/1447 (0.21%)	1/995 (0.10%)	2.84 (0.26–30.59)
								*BRCA2*	Any pathogenic variant	Not stated	9/1447 (0.62%)	3/995 (0.30%)	1.91 (0.48–7.59)
	Uganda	2010–2016	Cases vs controls from the same population	Cases: men with prostate cancer from 13 clinics in Uganda. Controls: patients recruited from non-urologic clinics in Uganda.	Mean: 70.77	Mean: 65.04	Covariate adjustment for age and genetic ancestry	*BRCA1*	Any pathogenic variant		2/651 (0.31%)	1/486 (0.21%)	1.11 (0.09–13.54)
								*BRCA2*	Any pathogenic variant	Not stated	12/651 (1.84%)	1/486 (0.21%)	10.30 (1.28–82.58)
Momozawa [22]	Japan, BioBank Japan	2003–2018	Cases vs controls from the same population	Cases: unselected men with PCa from a nationwide hospital-based biobank. Controls: male non-cancer patients from the same biobank older than 60 and with no personal or family history of cancer in first- or second-degree relatives.	Mean: 71.0	Mean: 70.4	None, but cases and controls were of comparable ages	*BRCA1*	Any pathogenic variant		14/7636 (0.18%)	10/12366 (0.08%)	2.27 (0.94–5.71)
								*BRCA2*	Any pathogenic variant	Not specified	83/7636 (1.09%)	24/12366 (0.19%)	5.65 (3.55–9.32)
Oak [24]	USA (predominantly European ancestry), The Cancer Genome Atlas	2005–2013	Cases vs controls from the same population	Cases: men with PCa from a nationwide biobank. Controls: patients with non-prostate cancers from the same biobank.	Not stated	Not stated	Covariate adjustment for age and genetic ancestry	*BRCA1*	Any pathogenic variant		3/409 (0.73%)	Not stated (total: 7711 non-PCa patients)	2.20 (0.62–7.83)
Wokolorczyk [25]	Poland	2000–2017	Cases vs controls from the same population	Cases: men with PCa who had a family history of PCa in first- or second-degree relatives (three or more relatives with PCa, or two affected relatives of whom at least one was diagnosed before age 60). Controls: participants in an unrelated population-based study.	Mean: 61.6	Mean: 59.4	None, but cases and controls were of comparable ages	*BRCA1*	Any pathogenic variant		5/390 (1.28%)	1/308 (0.32%)	4.0 (0.5–34.3)
								*BRCA2*	Any pathogenic variant	0/4 (0%)	4/390 (1.03%)	0/308 (0.00%)	--
Nguyen-Dumont [26]	Australia	Not stated	Cases vs controls from the same population	Cases: men with aggressive prostate cancer (T4, M1, N1 or Gleason score≥8) from four cohort studies and case series. Controls: male participants in an unrelated trial.^c^	Median: 65–69	Not stated (all ≥70)	Covariate adjustment for age	*BRCA1*	Any pathogenic variant		5/833 (0.60%)	10/5356 (0.19%)	2.9 (0.66–12.5)
								*BRCA2*	Any pathogenic variant	6/21 (29%)	19/833 (2.28%)	17/5356 (0.32%)	3.9 (1.1–13)

*PCa* prostate cancer, *PV* pathogenic variant, *OCCR* ovarian cancer cluster region, *OR* odds ratio, *CI* confidence interval.

^a^When available, the table includes adjusted odds ratio estimates (as indicated in the “age-adjustment” field). Otherwise, the reported unadjusted odds ratio estimates by each study are included. For studies that did not report odds ratios, unadjusted odds ratio estimates calculated from the frequencies of case and control PV carriers were used in the meta-analysis (not shown in this descriptive table but included in the forest plots).

^b^Reported on both *BRCA1* and *BRCA2*, but is not included in the *BRCA1* meta-analysis due to observing no *BRCA1* PVs in the cases which hence did not enable estimation of a 95% CI for the RR.

^c^The main analysis in this study compared cases with aggressive prostate cancer to a combined comparison group comprising cases with non-aggressive prostate cancer and unaffected men. The meta-analysis includes the supplementary analysis of cases with aggressive prostate cancer versus unaffected men.

**Table 2:** Cohort studies.

Publication	Population, dataset	Period	Study design	Selection	Average age	Age-adjustment	Gene	Considered PVs	% PVs located in *BRCA2* OCCR	*N*	RR (95% CI)^a^
BCLC [2]	Europe and North America (predominantly European ancestry), BCLC	Not stated	Kin-cohort	Families with a history of breast and/or ovarian cancer and at least one known *BRCA2* carrier, recruited through genetics clinics.	Not stated	Comparison to age-specific population incidence	*BRCA2*	Any pathogenic variant	Not specified	29 PCa in male *BRCA2* carriers from 173 breast-ovarian cancer families	4.65 (3.48–6.22)
Thompson [5]	Europe and North America (predominantly European ancestry), BCLC	Until 1999	Kin-cohort	Families with a history of breast and/or ovarian cancer and at least one known *BRCA1* carrier, recruited through genetics clinics.	Not stated	Comparison to age-specific population incidence	*BRCA1*	Any pathogenic variant		11 PCa in male *BRCA1* carriers and 7 PCa in non-carriers from 699 families	1.07 (0.75–1.54)
van Asperen [8]	The Netherlands, GEO-HEBON	1998–2003	Kin-cohort	Relatives of breast or ovarian cancer cases who had undergone breast and ovarian cancer counselling in 8 clinics in the Netherlands and who tested positive for *BRCA2* PVs.	Not stated	Comparison to age-specific population incidence	*BRCA2*	Any pathogenic variant	92/139 (66%) of family PVs	24 PCa in 803 men from 139 *BRCA2* families	2.5 (1.6–3.8)
Risch [9]	Canada (predominantly European ancestry)	1995–1999	Kin-cohort	Probands with ovarian cancer who were identified through a cancer register and who provided cancer family history information.	Not stated	Comparison to age-specific population incidence	*BRCA1*	Any pathogenic variant		4 PCa in 75 *BRCA1* families and 89 PCa in 1042 non-carrier families	0.65 (0.051–8.3)
							*BRCA2*	Any pathogenic variant	27/54 (50%) of family PVs	9 PCa in 54 *BRCA2* families and 89 PCa in 1042 non-carrier families	2.7 (1.1–7.1)
Moran [16]	UK	1996 and after	Family-based retrospective cohort	Families seeking genetic counselling in two clinics in England from which at least one individual tested positive for *BRCA1/2* PVs.	Not stated	Comparison to age-specific population incidence	*BRCA1*	Any pathogenic variant		6.1 standardised PCa observations in male *BRCA1* carriers from 268 *BRCA1* families	1.0 (0.4–2.3)
							*BRCA2*	Any pathogenic variant	90/222 (41%) of family PVs^b^	31.7 standardised PCa observations in male *BRCA2* carriers from 222 *BRCA2* families	6.3 (4.3–9.0)
Page [20]	International (predominantly European ancestry), IMPACT	2005–2015	Prospective screening cohort	*BRCA1/2*-positive and *BRCA1/2*-negative men aged 40–69 from families with *BRCA1/2* PV, recruited through 65 centres in 20 countries.	Not stated (median enrolment age across all participants: 54)	Covariate adjustment for age, ethnicity and country	*BRCA1*	Any pathogenic variant		19 PCa in 919 *BRCA1* carriers, 14 PCa in 709 non-carriers	1.36 (0.75–2.45)
							*BRCA2*	Any pathogenic variant	42%^b^	57 PCa in 902 *BRCA2* carriers, 20 PCa in 497 non-carriers	1.95 (1.06–3.56)
Nyberg [23]	UK and Ireland, EMBRACE	1999–2016	Prospective cohort	Unaffected men with *BRCA1/2* PVs recruited nationwide through genetics centres and followed prospectively for PCa development.	Median: 54.0 *BRCA1*; 51.4 *BRCA2*	Comparison to age-specific population incidence	*BRCA1*	Any pathogenic variant		16 PCa in 376 *BRCA1* carriers	2.35 (1.43–3.88)
							*BRCA2*	Any pathogenic variant	178/445 (40%)	26 PCa in 447 *BRCA2* carriers	4.45 (2.99–6.61)

*PCa* prostate cancer, *PV* pathogenic variant, *OCCR* ovarian cancer cluster region, *RR* relative risk, *CI* confidence interval.

^a^In all studies except Page et al. [20], the RR represents the estimated standardised incidence ratio, comparing pathogenic variant carriers to age-specific population cancer incidences. Page et al. [20] adjusted for age, ethnicity and country.

^b^Provided by the study authors on request.

**Fig. 2:**
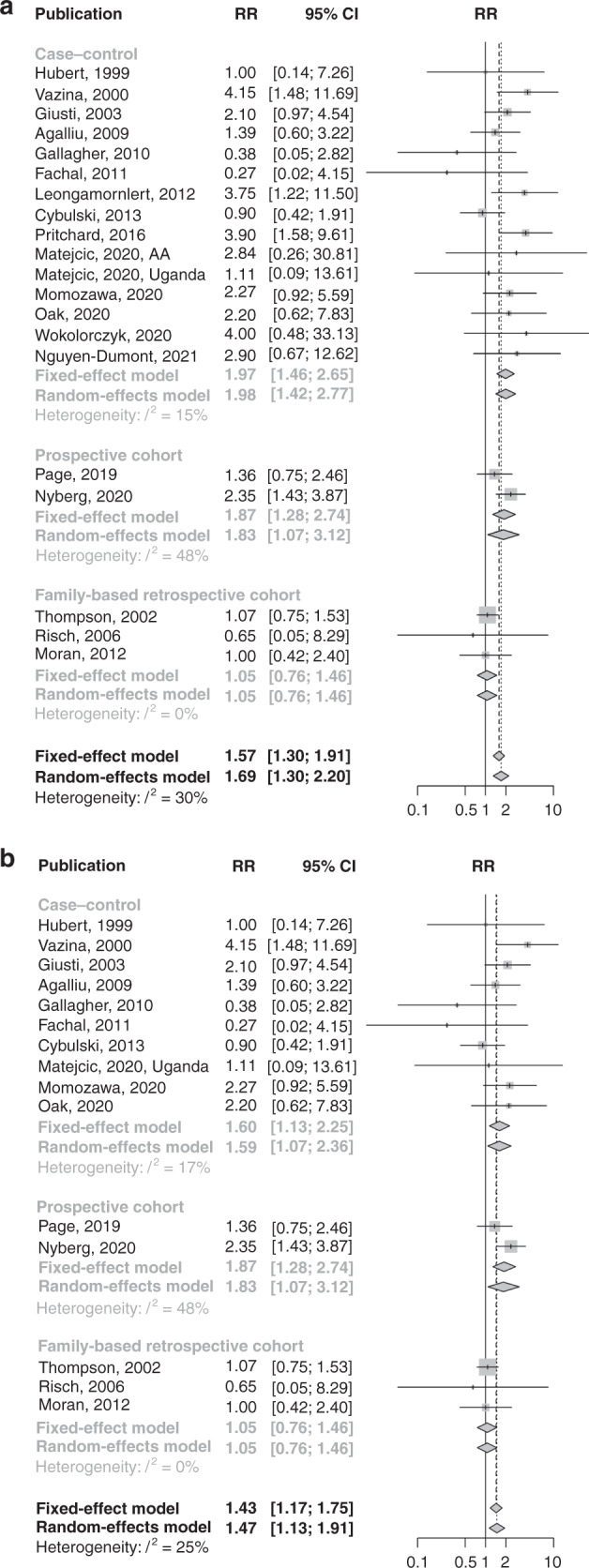
Forest plots of overall *BRCA1* RR estimates. **a** All initially considered studies; **b** after restriction to studies unselected for age at diagnosis, family history or aggressive disease.

**Fig. 3:**
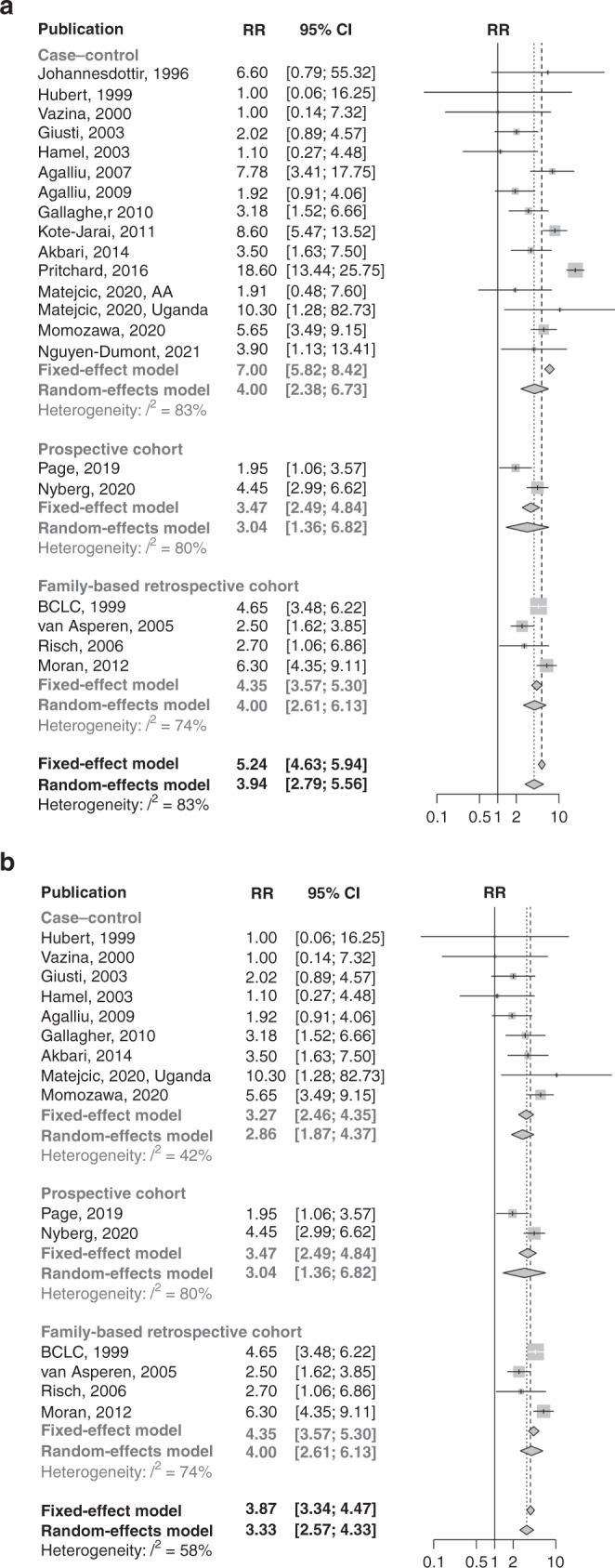
Forest plots of overall *BRCA2* RR estimates. **a** All initially considered studies; **b** after restriction to studies unselected for age at diagnosis, family history or aggressive disease.

The reported RR estimates showed a high degree of variability, particularly those for *BRCA2* carriers (*BRCA1*: *I*^2^ = 30%, *BRCA2*: *I*^2^ = 83%; Figs. 2 and 3). The funnel plots indicated both high and low RR estimates as outliers and that smaller *BRCA2* studies generally reported lower RR estimates than larger studies. However, there was no statistically significant funnel plot asymmetry (Supplementary Figs. S1 and S2).

The RR estimates from studies that selected participants for PCa diagnosis at a young age, PCa family history or aggressive PCa were higher than estimates from studies in unselected participants (*BRCA1*: test for subgroup differences, *P* = 0.056, *BRCA2*: test for subgroup differences, *P* < 0.001; Supplementary Table S2). We restricted the main meta-analysis to studies unselected for age at PCa diagnosis, PCa family history or aggressive PCa, but separately analysed these subgroups. Table 3 summarises the pooled RR estimates from the further restrictions, subgroup analyses and adjustments made in the meta-analysis.

**Table 3:** Heterogeneity and pooled RR estimates by study subgroups.

Gene, age group	Subgroup	Selection	No. of studies	Fixed-effect pooled RR (95% CI)	Random-effect pooled RR (95% CI)	*I*^*2*^
*BRCA1*, overall	All	All estimates	20	1.57 (1.30–1.91)	1.69 (1.30–2.20)	30%
	Studies unselected for age, aggressive prostate cancer, or family history of prostate cancer	All estimates	15	1.43 (1.17–1.75)	1.47 (1.13–1.91)	25%
	Studies unselected for age, aggressive prostate cancer, or family history of prostate cancer; and, that did not use historical controls	All estimates	13	1.32 (1.07–1.64)	1.33 (1.05–1.69)	8%
		All estimates: EMBRACE prospective RR estimate adjusted for potential screening effects^a^	13	1.18 (0.95–1.46)	1.18 (0.95–1.46)	0%
		Ashkenazi ancestry	3	1.12 (0.55–2.31)	1.12 (0.55–2.31)	0%
		Non-Ashkenazi European ancestry	8	1.30 (1.03–1.64)	1.30 (0.95–1.79)	30%
		Non-Ashkenazi European ancestry: EMBRACE prospective RR estimate adjusted for potential screening effects^a^	8	1.13 (0.89–1.44)	1.13 (0.89–1.44)	0%
		African ancestry	1	1.11 (0.09–13.61)	1.11 (0.09–13.61)	--
		Asian ancestry	1	2.27 (0.92–5.59)	2.27 (0.92–5.59)	--
*BRCA2*, overall	All	All estimates	21	5.24 (4.63–5.94)	3.94 (2.79–5.56)	83%
	Studies unselected for age, aggressive prostate cancer, or family history of prostate cancer	All estimates	15	3.87 (3.34–4.47)	3.33 (2.57–4.33)	58%
	Studies unselected for age, aggressive prostate cancer, or family history of prostate cancer; by ethnicity	Ashkenazi ancestry: all estimates	6	2.08 (1.38–3.12)	2.08 (1.38–3.12)	0%
		Non-Ashkenazi European ancestry: all estimates	7	4.07 (3.45–4.80)	3.69 (2.71–5.04)	66%
		Non-Ashkenazi European ancestry: excluding two outliers^b^	5	3.89 (3.20–4.73)	3.71 (2.82–4.89)	39%
		Non-Ashkenazi European ancestry: excluding three outliers^c^	4	4.35 (3.50–5.41)	4.35 (3.50–5.41)	0%
		African ancestry: all estimates	1	10.30 (1.28–82.73)	10.30 (1.28–82.73)	--
		Asian ancestry: all estimates	1	5.65 (3.49–9.15)	5.65 (3.49–9.15)	--
	Studies unselected for age, aggressive prostate cancer, or family history of prostate cancer; by the proportion of PVs located in the OCCR	≥50% OCCR PVs: all estimates	8	2.30 (1.74–3.06)	2.30 (1.74–3.06)	0%
		≥50% OCCR PVs: using separate OCCR estimates when available	9	2.15 (1.61–2.88)	2.15 (1.61–2.88)	0%
		OCCR PVs^d^	8	2.10 (1.55–2.86)	2.10 (1.55–2.86)	0%
		<50% OCCR PVs: all estimates	4	4.74 (3.81–5.91)	4.38 (2.83–6.77)	73%
		<50% OCCR PVs: excluding one outlier^e^	3	5.43 (4.29–6.87)	5.43 (4.29–6.87)	0%
		<50% OCCR PVs: using separate non-OCCR estimates when available	4	5.65 (4.49–7.12)	5.65 (4.49–7.12)	0%
		Non-OCCR PVs^d^	2	5.06 (3.48–7.36)	4.93 (3.10–7.82)	28%
		Proportion of PVs located in OCCR not determinable: all estimates	3	4.55 (3.48–5.95)	4.55 (3.48–5.95)	0%
*BRCA1*, age <65 years	All	All estimates	4	2.21 (1.47–3.30)	2.19 (1.21–3.98)	47%
		Excluding one outlier^f^	3	2.52 (1.64–3.87)	2.59 (1.58–4.24)	19%
		EMBRACE prospective RR estimate adjusted for potential screening effects^a^	4	1.79 (1.17–2.72)	1.78 (1.12–2.85)	14%
		Studies that did not use historical controls or external population estimates	3	2.04 (1.32–3.14)	1.92 (0.94–3.92)	57%
		Excluding one outlier^f^, EMBRACE prospective RR estimate adjusted for potential screening effects^a^, studies that did not use historical controls or external population estimates	2	1.78 (1.09–2.91)	1.78 (1.09–2.91)	0%
*BRCA1*, age ≥65 years	All	All estimates	3	1.18 (0.83–1.70)	1.43 (0.71–2.87)	65%
		Excluding one outlier^f^	2	1.09 (0.75–1.59)	1.21 (0.55–2.62)	73%
		EMBRACE RR estimate adjusted for potential screening effects^a^	3	1.01 (0.70–1.45)	1.10 (0.65–1.86)	39%
		Studies that did not use historical controls or external population estimates	3	1.18 (0.83–1.70)	1.43 (0.71–2.87)	65%
		Excluding one outlier^f^, EMBRACE prospective RR estimate adjusted for potential screening effects^a^, studies that did not use historical controls or external population estimates	2	0.91 (0.62–1.33)	0.91 (0.62–1.33)	0%
*BRCA2*, age <65 years	All	All estimates	5	6.37 (4.81–8.43)	5.28 (3.10–9.00)	63%
	By ethnicity	Ashkenazi ancestry	1	1.58 (0.57–4.38)	1.58 (0.57–4.38)	--
		Non-Ashkenazi European ancestry	4	7.14 (5.33–9.56)	7.14 (5.33–9.56)	0%
*BRCA2*, age ≥65 years	All	All estimates	3	3.74 (2.82–4.96)	3.74 (2.82–4.96)	0%
	By ethnicity	Ashkenazi ancestry	1	2.63 (0.85–8.16)	2.63 (0.85–8.16)	--
		Non-Ashkenazi European ancestry	2	3.83 (2.86–5.12)	3.84 (2.84–5.18)	6%

*RR* relative risk, *CI* confidence interval, *PV* pathogenic variant, *OCCR* ovarian cancer cluster region.

^a^Using a 6 month landmark and compared to population incidences adjusted by a factor of 1.6 [23].

^b^Excluding the studies by Page and coworkers [20] and Moran and coworkers [16].

^c^Excluding the studies by Page and coworkers [20], Moran and coworkers [16] and van Asperen and coworkers [8].

^d^Restricted to studies that reported separate RR estimates for OCCR and non-OCCR PVs, or where all reported PVs were located in the OCCR.

^e^Excluding the study by Page and coworkers [20].

^f^Excluding the study by Agalliu and coworkers [11].

### *BRCA1*

Studies on *BRCA1* carriers that relied on historical controls reported higher RR estimates than other studies (test for subgroup differences, *P* = 0.044; Supplementary Table S3).

#### *BRCA1*: studies without historical controls

Restricted to studies of *BRCA1* carriers that did not use historical controls, the heterogeneity between estimates was low (*I*^2^ = 8%; Supplementary Figs. S3 and S4; Supplementary Table S4). A leave-one-out analysis identified the prospective EMBRACE study [23] as a high outlier (*P* = 0.013; Supplementary Table S5). The EMBRACE study reported a screening-bias-corrected estimate; [23] Table 3 shows the pooled RR when this estimate was used instead (Supplementary Figs. S3 and S4 and Table 3).

### *BRCA2*

*BRCA2* studies in Ashkenazi Jewish men reported lower RR estimates than studies in other populations (test for subgroup differences, *P* = 0.011). The RR estimates were lower in studies where ≥50% of the reported PVs were located in the OCCR (test for subgroup differences, *P* = 0.002; Supplementary Table S3).

#### *BRCA2*: prostate cancer risk by ethnicity

Table 3 shows pooled RR estimates based on studies in Ashkenazi Jewish populations (Supplementary Figs. S5 and S6), where the heterogeneity between estimates was low (*I*^2^ = 0%; Supplementary Tables S6 and S7).

For studies of *BRCA2* carriers in non-Ashkenazi European ancestry populations (Supplementary Figs. S5 and S6), the heterogeneity between estimates was high (*I*^2^ = 66%). A leave-one-out analysis identified three outliers (Supplementary Table S7): a UK family-based retrospective cohort study (*P* = 0.010) [16], the IMPACT screening trial (*P* = 0.013) [20], and a Dutch kin-cohort study (*P* = 0.017) [8]. Table 3 shows pooled RR estimates after excluding these studies. Notably, the main estimate from the EMBRACE study [23] was not an outlier among the estimates for *BRCA2* carriers (*P* = 0.6), and if instead a screening-effect-adjusted estimate was used, the RR estimate was an outlier and significantly lower than the other estimates (*P* = 0.025).

#### *BRCA2*: prostate cancer risk by pathogenic variant location

Table 3 shows pooled RR estimates in studies split by OCCR proportion, before and after exclusion of the IMPACT study [20] which was a low outlier among studies with <50% OCCR PVs (*P* = 0.002; Supplementary Figs. S7 and S8; Supplementary Tables S8 and S9), and after restriction to the available OCCR- or non-OCCR-specific estimates.

Furthermore, a meta-regression model showed a trend towards linearly decreasing log-RR estimates with the increasing proportion of OCCR PVs in a study (*P* < 0.001). The regression model had low residual heterogeneity (*I*^2^ = 5%), and predicted RRs of 2.31 (95% CI 2.20–2.42) from studies with 100% OCCR PVs and 6.50 (95% CI 6.14–6.87) from studies with 0% OCCR PVs (Supplementary Fig. S9).

### Prostate cancer risk by age group

Supplementary Figs. S10 and S11 show all reported RR estimates by the age cutpoints used to define age groups. Restricted to RR estimates by age groups younger or older than 65 years, the RRs were heterogeneous for both *BRCA1* (age <65 years *I*^2^ = 47%, age ≥65 years *I*^2^ = 65%; Supplementary Figs. S12 and S13) and *BRCA2* carriers (age <65 years *I*^2^ = 63%, age ≥65 years *I*^2^ = 0%; Supplementary Figs. S14 and S15).

#### *BRCA1*

The age-specific estimates from a large international kin-cohort study [5] were somewhat lower at age≥65 years than estimates from other studies (age <65 years *P* = 0.4, age ≥65 years *P* = 0.019; Supplementary Tables S10 and S11). However, we could not identify any likely methodological explanation for this outlying estimate and therefore retained the study. The age-specific RR estimates from one case–control study in Ashkenazi Jewish men [11] were somewhat lower at younger ages and somewhat higher at older ages than estimates from other studies (age <65 years *P* = 0.073, age ≥65 years *P* = 0.15; Supplementary Table S11) and the RR estimates from the EMBRACE study [23] were somewhat higher than estimates from other studies at both younger and older ages (age <65 years *P* = 0.14, age ≥65 years *P* = 0.11; Supplementary Table S11), but these differences were not significant. Table 3 shows the results when excluding the study in Ashkenazi men, including screening-effect-adjusted estimates from EMBRACE, or restricting to studies that did not rely on external population frequency estimates.

#### *BRCA2*

The RR estimate for younger *BRCA2* carriers from one study of Ashkenazi Jewish men [11] was a low outlier (age <65 years *P* = 0.005, age ≥65 years *P* = 0.5; Supplementary Fig. S14; Supplementary Tables S12 and S13). Table 3 shows pooled RR estimates by age group before and after excluding this study.

### Prostate cancer risk by family history of prostate cancer

The pooled RR estimate for *BRCA1* carriers with PCa family history was 2.79 (95% CI 1.33–5.88; *I*^2^ = 0%). Only one study reported a RR specifically for *BRCA2* carriers with a family history, of 7.31 (95% CI 3.40–15.7).

### Risk of aggressive prostate cancer

The pooled random-effects RRs of aggressive PCa (any definition) were 1.98 (1.35–2.90; *I*^2^ = 0%) for *BRCA1* carriers and 6.08 (3.44–10.8; *I*^2^ = 82%) for *BRCA2* carriers (Supplementary Fig. S16). For *BRCA2* carriers, the RR estimates differed significantly by the definition of aggressive PCa (*P* < 0.001), with higher RR estimates reported for metastatic or Gleason score≥8 PCa than Gleason score≥7 PCa. For *BRCA1*, there was no significant heterogeneity by the definition of aggressive PCa (*P* = 0.3). Restricted to estimates of the RR of Gleason score ≥7 PCa, the pooled random-effects RRs were 1.59 (95% CI 1.02–2.49; *I*^2^ = 0%) for *BRCA1* carriers and 4.94 (95% CI 3.51–6.96; *I*^2^ = 0%) for *BRCA2* carriers.

## Discussion

A wide range of PCa RR estimates have been reported for *BRCA1* and *BRCA2* carriers. The results of this meta-analysis suggest that the heterogeneity may in part be explained by selection for age, family history or aggressive disease, and study-level differences in the age and ethnic ancestry composition of the study participants, the reliance of some studies on historical controls, and the proportion of the studied *BRCA2* carriers who have PVs within the OCCR.

The pooled RR estimates indicate that male *BRCA2* carriers are at higher than population risk of PCa at all ages, whereas *BRCA1* carriers may be at somewhat increased risk with the increased risk restricted to younger ages. Based on the most restrictive inclusion criteria considered, the overall random-effects RR estimates were 2.08 (95% CI 1.38–3.12) for Ashkenazi Jewish *BRCA2* carriers and 4.35 (95% CI 3.50–5.41) for non-Ashkenazi European ancestry *BRCA2* carriers. This heterogeneity in *BRCA2* PCa risks by ethnicity indicates the need for further research to explore ethnicity-specific risk estimates for male *BRCA2* carriers. The reported RRs for African and Asian ancestry *BRCA2* carriers were similar to those for non-Ashkenazi European ancestry men, but this was based on a small number of studies and should be interpreted with caution. However, even if the RRs are similar, this would translate to different absolute risks for *BRCA2* carriers by ethnicity, because the baseline population risks differ between ethnic groups [43, 44]. For *BRCA1* carriers, there was no significant difference in reported RRs by ethnicity and the overall RR was estimated to be 1.18 (95% CI 0.95–1.46). For both *BRCA1* and *BRCA2* carriers, the reported RRs were higher at younger ages. Based on the most restrictive inclusion criteria, the estimated age-specific RRs applicable to non-Ashkenazi European ancestry men were 7.14 (95% CI 5.33–9.56) at ages <65 and 3.84 (95% CI 2.84–5.18) at ages ≥65 years for *BRCA2* carriers and 1.78 (95% CI 1.09–2.91) at ages <65 and 0.91 (95% CI 0.62–1.33) at ages ≥65 years for *BRCA1* carriers.

The reported overall RR estimates for *BRCA2* carriers were lower from studies where a majority of the *BRCA2* PVs were located in the OCCR (pooled RR = 2.30, 95% CI 1.74–3.06). The meta-regression showed a trend towards decreasing RRs with increasing study-level proportions of PVs located in the *BRCA2* OCCR, consistent with the observations that carriers of *BRCA2* PVs within the OCCR have a lower risk of PCa than other *BRCA2* PV carriers [8, 23, 29–32]. The Ashkenazi *BRCA2* studies reported exclusively on the Ashkenazi founder PV c.5946delT that is located in the OCCR, and the RRs from these studies (pooled RR = 2.08, 95% CI 1.38–3.12) were comparable with the RRs reported from studies in non-Ashkenazi European ancestry populations where the majority of participants had PVs located in the OCCR (pooled RR = 2.53, 95% CI 1.71–3.75). Hence, as has previously been suggested [11], it is possible that the lower PCa risks observed for Ashkenazi *BRCA2* carriers [3, 4, 6, 7, 11, 12, 30, 33, 45] is explained by risk variation by the location of PVs within the *BRCA2* gene.

By contrast, there was no significant variation in the reported overall *BRCA1* RR estimates by the ethnic ancestry of the study participants. The studies in Ashkenazi Jewish men reported exclusively on the two Ashkenazi founder PVs c.68_69delAG and/or c.5266dupC. A lack of variation in the PCa risk by specific founder PVs is consistent with previous findings of a lack of significant variation by the location of PVs within *BRCA1* [31]. Moreover, the reported RR estimates were higher from two studies that compared Israeli PCa patients to controls from previous studies of US Ashkenazi individuals [4, 6]. The use of cases and controls from different settings and time periods make the studies susceptible to bias from population stratification, and place- and time-specific differences in e.g. opportunistic screening rates. Only one study in Ashkenazi Jewish *BRCA1* carriers had reported age-specific RR estimates [11], and these were somewhat lower for younger carriers and somewhat higher for older carriers compared to estimates from studies in non-Ashkenazi European ancestry populations. This study was however limited by the use of a self-selected sample and ascertainment bias may be likely. Hence, the finding may not be inconsistent with the finding of no significant differences by ethnicity in the meta-analysis of overall RR estimates for *BRCA1* carriers.

The RR estimates from the EMBRACE study were identified as high outliers among the *BRCA1* but not the *BRCA2* estimates. The EMBRACE study was limited by potential confounding by screening effects [23]. *BRCA2* PVs are associated with a more aggressive PCa phenotype than *BRCA1* PVs [11, 12, 20, 23, 46], and the results may hence reflect that *BRCA2* carriers are more likely than *BRCA1* carriers to have clinically significant PCa which is diagnosed regardless of screening. When we instead included *BRCA1* RR estimates from a sensitivity analysis that adjusted for potential screening effects, these RR estimates were consistent with those reported in other studies. The IMPACT screening trial reported an RR estimate for *BRCA2* carriers that was significantly lower than estimates from other studies. Enhanced screening makes early diagnoses of indolent tumours likely in the trial arms. Hence, bias towards the null may be expected compared to the risk for the average *BRCA1/2* carrier in the population, if overdiagnosis rates are similar in the carriers and non-carriers.

One case–control study included only cases with a family history of PCa and an unselected control group, and did not adjust for this family history-based ascertainment [25]. This is likely to lead to higher RR estimates compared to RRs based on case–control studies of unselected cases, because of likely enrichment of PCa PVs in subjects from PCa families. Although such designs may provide valid tests of association, they can lead to biased RR estimates [47]. Two family-based retrospective cohort studies in relatives of breast or ovarian cancer cases reported estimates that were significantly higher [16] or lower [8] than estimates from other studies. Assuming that no other shared genetic and familial risk factors besides *BRCA1/2* PVs exist between PCa, breast and ovarian cancer, such ascertainment should in principle not introduce ascertainment bias. However, given the excess breast cancer risk in relatives of PCa cases [48] and the established associations between *BRCA1/2* PVs and PCa, it cannot be ruled out that testing for *BRCA1/2* PVs in individuals with breast cancer may in some instances have been influenced by the presence of PCa cases in the family. If so, failing to adjust for the PCa events that determined the ascertainment would bias the resulting PCa RRs away from the null. One study included biopsy-negative individuals as controls [18], one study used controls who had other cancers [24] and two studies used controls identified in healthcare settings [21, 22]. Such control selection might bias the corresponding RR estimates if the PV frequency among the controls differs systematically from the population. However, the meta-analysis did not suggest significant differences between these estimates and estimates from other studies.

The systematic review and meta-analysis has a number of strengths. Since the most recent previous systematic review and meta-analysis [27], seven studies [20–26] have been published, including two prospective studies [20, 23] and studies in African [21] and Asian [22] ancestry populations. By incorporating these studies, we update the available evidence. Furthermore, our meta-analysis expanded on previous meta-analyses by exploring variability in risks, which identified several possible explanatory factors for the heterogeneity between studies. We have provided estimates that synthesise all available data on the RRs of PCa for male *BRCA1* and *BRCA2* carriers.

The systematic review and meta-analysis also has limitations. Publication bias and selective reporting of significant outcomes within studies may bias meta-analysis estimates [49]. Because only a subset of the studies reported RRs by age, family history and PV location, and RRs of aggressive PCa, such bias cannot be ruled out. Funnel plots for the age-specific estimates showed no clear asymmetry, indicating that selective reporting is less likely. Another limitation is the potential overlap between the participants of different studies. As noted above some studies used the same historical controls, and the *BRCA1/2* carrier participants partially overlapped between EMBRACE [23] and IMPACT [20]. This invalidates the assumption that the RRs are estimated based on independent samples, which may bias the pooled RR estimates and underestimate the width of the associated CIs. The meta-analysis of *BRCA2* OCCR PVs was limited by a lack of separate estimates of the risks associated with OCCR and non-OCCR PVs. The analysis predominantly relied on study-level data on the proportion of reported PVs that were located within the OCCR. For some studies, this proportion was based on the family-level rather than the individual-level PV distribution. However, despite these limitations, the resulting RR estimate (pooled RR = 2.30, 95% CI 1.74–3.06) was consistent with the eight separate estimates reported for OCCR PVs (pooled RR = 2.10, 95% 1.55–2.86). Risk variation by the OCCR was however not present when split by age group. This might be due to the use of the study-level proportion of OCCR PV carriers, which may be a poor proxy for the proportion of OCCR PV carriers within age-stratified subgroups of the study participants. These study-level subgroup analyses are hypothesis-generating and larger studies are needed to estimate the age-specific risk associated with specific subgroups of *BRCA1/2* carriers based on individual-level data, e.g. by ethnic ancestry and PV location. Finally, the literature search and review was performed by a single reviewer rather than several reviewers, and although the review assessed sources of study-specific bias, it did not use a standardised rating scale.

### Conclusion

This meta-analysis has identified several potential effect modifiers that may guide future studies, and has provided pooled RR estimates, overall and by age group, of the risk of PCa for male *BRCA1* and *BRCA2* carriers that incorporate the current accumulated evidence. These risk estimates will be informative for the genetic counselling of male *BRCA1* and *BRCA2* carriers.

## Supplementary information

Supplementary file

## Data Availability

The literature review and meta-analysis datasets generated and analysed during the current study are available from the corresponding author on reasonable request.
