# Supplementary material for: BRCA1 and BRCA2 pathogenic variants and prostate cancer risk: systematic review and meta-analysis
Source: Br J Cancer. 2021 Dec 28;126(7):1067–81. doi: 10.1038/s41416-021-01675-5 (PMC8979955; doi:10.1038/s41416-021-01675-5)
Supplement: Supplementary file 1 — Supplementary file [file 41416_2021_1675_MOESM1_ESM.pdf]

BRCA1 and *BRCA2* pathogenic variants and prostate cancer risk: systematic review and meta-analysis

Supplementary material

Tommy Nyberg, Marc Tischkowitz, Antonis C. Antoniou

A.1 Literature search

The following search query was used to search the PubMed, Embase, MEDLINE and Cochrane Library databases: "((BRCA OR BRCA1 OR BRCA2) AND (mutation OR mutations OR variant OR variants OR polymorphism OR polymorphisms OR SNP OR SNPs)) AND (prostate AND (cancer OR cancers OR carcinoma OR carcinomas OR neoplasm OR neoplasms OR tumour OR tumours OR tumor OR tumors OR malignancy OR malignancies)) AND (risk OR risks OR penetrance OR penetrances)".

Table S1 lists definitions of the study-level covariates considered in the meta-analysis.

A.2 Identified studies: Detailed description

The literature search identified a total of 2,547 entries. among which 503 publications reported original data. The first screening round identified 108 potentially relevant publications. After review of the full text of these articles, we identified a final set of 26 articles that reported on 27 studies that provided estimates of the RR of PCa for *BRCA1* (n=20) and/or *BRCA2* carriers (n=21; Figure 1).[1–26]

A.2.1 Case-control studies

Table 1 summarises the 20 studies that used a case-control study design (*BRCA1* n=15, *BRCA2* n=15).[1,3,4,6,7,10–15,17–19,21,22,24–26]

As detailed below, eight of these studies selected cases for young age at PCa diagnosis,[1,10,14,15] positive PCa family history,[25] or aggressive PCa.[19,21,26] The remaining 12 case-control studies were based on cases unselected for age at diagnosis, family history, and disease aggressiveness.[3,4,6,7,11–13,17,18,21,22,24]

Three studies used patients with other diseases than PCa as controls,[21,22,24] and the remaining 17 studies relied on control study participants from the general population or who were PSA test or biopsy negative for PCa.[1,3,4,6,7,10–15,17–19,21,25,26] Among the latter, three studies compared historical controls from previous studies of US Ashkenazi Jewish individuals with cases of Ashkenazi Jewish ancestry in other countries,[4,6,7] and four studies compared the PV carrier frequencies of cases with publicly available population carrier frequency estimates.[10,14,15,19]

A.2.2 Cohort studies

Table 2 summarises the data from cohort studies that reported PCa risks for *BRCA1/2* carriers.

Prospective cohort studies Two studies reported prospective RR estimates (*BRCA1* n=2, *BRCA2* n=2).[20,23] One was the IMPACT screening trial, that prospectively followed *BRCA1/2* carriers and a comparison group of confirmed *BRCA1/2* PV negative men from *BRCA1/2* carrier families. The participants in both groups received annual PSA screening to evaluate if carriers would benefit from screening.[20] The other study was the EMBRACE study.[23] This study prospectively followed PCa-free men identified in genetic counselling clinics for the development of PCa.

Family-based retrospective cohort studies Five studies were family-based retrospective cohort or kin-cohort studies,[2,5,8,9,16] in which family members of known PV carriers were retrospectively followed for the development of PCa. All of the family-based studies included families ascertained based on relatives diagnosed with breast and/or ovarian cancer.

A.2.3 Age selection

Four studies included only cases on the basis of a young age at PCa diagnosis,[1,10,14,15] and six studies provided separate RR estimates by age in addition to their primary RR estimates.[2,5,8,11,17,23] Seven studies reported RRs for ages below and/or above 65 years,[1,2,5,11,14,15,23] and the remaining studies used the age cutpoints 55 years,[10] 60 years[17] or 73 years.[8]

A.2.4 Family history selection

One study included only cases with positive PCa family history.[25] Two additional studies reported subgroup RR estimates for participants with positive family history.[17,23]

A.2.5 Aggressive PCa selection

Three studies selected for aggressive PCa: one study included only metastatic PCa cases,[19] one study included only PCa cases with lethal PCa, T4, or T3 and Gleason score≥8 PCa,[26] and one study included cases "overselected for high stage and Gleason score".[21] Seven additional studies reported separate RRs of aggressive PCa, in addition to their primary RR estimates.[11,12,17,18,22,23,25]

A.2.6 Pathogenic variant characteristics

Six studies in Ashkenazi populations included only the founder PVs c.68_69delAG and/or c.5266dupC in *BRCA1* and c.5946delT in *BRCA2*.[3,4,6,7,11,12] The number of *BRCA2* PVs located in the OCCR or a list of all included PVs which allowed classification of the proportion of PVs that were located within the OCCR were available for 17 studies.[1,3,4,6–12,14,16,19,20,22,23,26] Two of these studies reported separate RRs for *BRCA2* PVs within and outside the OCCR.[8,23]

A.3 Meta-analysis: Additional details

Table S2 shows the estimated heterogeneity and pooled RRs by subgroups of all included studies. Table S3 shows the estimated heterogeneity and pooled RRs by subgroups after restriction to studies where the participants were unselected for age at PCa diagnosis, PCa family history, or aggressive PCa. Figures S1 and S2 shows funnel plots of the RR estimates for *BRCA1* and *BRCA2* carriers, respectively, from all studies and after restriction to studies where the participants were unselected for age at PCa diagnosis, PCa family history, or aggressive PCa.

A.3.1 *BRCA1*: studies without historical controls

Figure S3 shows forest plots, and Figure S4 shows funnel plots, for *BRCA1* carriers after restriction to studies that did not rely on historical controls. Table S4 shows the corresponding estimated heterogeneity and pooled RRs by subgroups, and Table S5 shows the results from leave-one-out analyses.

A.3.2 *BRCA2*: prostate cancer risk by ethnicity

Figure S5 shows forest plots, and Figure S6 shows funnel plots, for *BRCA2* carriers by the study-level majority ethnicity of the study participants. Table S6 shows the corresponding estimated heterogeneity and pooled RRs by subgroups, and Table S7 shows the results from leave-one-out analyses.

A.3.3 *BRCA2*: prostate cancer risk by pathogenic variant location

Figure S7 shows forest plots, and Figure S8 shows funnel plots, for *BRCA2* carriers by whether a majority of the studies' reported PVs were located in the OCCR. Table S8 shows the corresponding estimated heterogeneity and pooled RRs by subgroups, and Table S9 shows the results from leave-one-out analyses. Figure S9 shows the results from a meta-regression model, where the reported RRs for *BRCA2* carriers were regressed on the study-level proportion of participants who had PVs within the OCCR.

A.3.4 Prostate cancer risk by age group

Figure S10 shows forest plots of the estimates by the age cutpoints used in the studies. Figure S12 shows forest plots, and Figure S13 shows funnel plots, for *BRCA1* carriers by age groups younger or older than 65 years. Table S10 shows the corresponding estimated heterogeneity and pooled RRs by subgroups, and Table S11 shows the results from leave-one-out analyses.

Figure S11 shows forest plots of the estimates by all age cutpoints used in the studies. Figure S14 shows forest plots, and Figure S15 shows funnel plots, for *BRCA2* carriers by age groups younger or older than 65 years. Table S12 shows the corresponding estimated heterogeneity and pooled RRs by subgroups, and Table S13 shows the results from leave-one-out analyses.

A.3.5 Risk of aggressive prostate cancer

Figure S16 shows estimates of aggressive PCa, by the definition of aggressive PCa.

Table S1: Definitions of the study-level characteristics used in the *BRCA1/2* meta-analysis. Abbreviations: PV, pathogenic variant. OCCR, ovarian cancer cluster region.

**Study-level characteristic**	**Definition**	**Categories**
Study design	Study design as determined from the publication.	Case-control.<br>Prospective cohort.<br>Family-based retrospective cohort.
Ethnicity	The majority ethnic ancestry of the study participants.	Ashkenazi Jewish.<br>Non-Ashkenazi Jewish European ancestry.<br>African ancestry.<br>Asian ancestry.
Age adjustment	The approach used to adjust for any age differences between comparison groups.	Age differences adjusted for or addressed through study design: Matching or covariate adjustment for age, or comparison to age-specific population incidences.<br>No age adjustment but age distribution was similar between the comparison groups: <5 years difference in the mean age, if reported, or otherwise median age, if reported, between the comparison groups; or, if ages were not reported, use of screening-positive and screening-negative cases and controls (which makes a similar age distribution likely).<br>No age adjustment.
Case/participant selection	Selection criteria for cases (for case-control studies) or participants (for cohort studies).	Selected for young age at prostate cancer onset: Selection of only (or enrichment for) cases or participants younger than a specified age threshold.<br>Selected for aggressive disease: Selection of only (or enrichment for) cases who fulfilled specified criteria for aggressive prostate cancer.<br>Selected for family history of prostate cancer: Selection of only (or enrichment for) cases or participants with a positive family history of prostate cancer.<br>Unselected for age, aggressive prostate cancer and family history.
Control selection	Selection criteria for controls (case-control studies).	Non-prostate cancer patients: Use of patient controls who had other diseases than prostate cancer.<br>Population controls: Use of unaffected controls from the general population.
Historical or external controls	Use of control data from historical studies or external sources (case-control studies).	Historical controls or external population estimates: Comparison to historical controls from previous studies in a different population setting and/or time period than the cases, or to publicly available population carrier frequencies.<br>Controls from the same population as the cases.
Proportion of *BRCA2* PVs located in OCCR	The proportion of the observed *BRCA2* PVs in cases (in case-control studies), participating individuals (in prospective cohort studies) or families (in family-based retrospective cohort studies) that were located within the wide definition OCCR (c.2831 to c.6401).	$\geq$50% OCCR PVs: A majority ($\geq$50%) of the observed PVs were located within the OCCR.<br><50% OCCR PVs: A minority (<50%) of the observed PVs were located within the OCCR.<br>Not determinable: Insufficient information were reported to classify which proportion of the observed PVs were located in the OCCR.

Table S2: Heterogeneity and pooled RR estimates by study subgroups.
Abbreviations: RR, relative risk. CI, confidence interval. PV, pathogenic variant. OCCR, ovarian cancer cluster region.

Gene	Covariate	Subgroup	No. studies	Fixed effects pooled RR (95% CI)	Random effects pooled RR (95% CI)	I^2	p-value[a]
BRCA1	Overall	All estimates	20	1.57 (1.30-1.91)	1.69 (1.30-2.20)	30%	
	Study design	Case-control	15	1.97 (1.46-2.65)	1.98 (1.42-2.77)	15%	0.022
		Prospective cohort	2	1.87 (1.28-2.74)	1.83 (1.07-3.12)	48%	
		Family-based retrospective cohort	3	1.05 (0.76-1.46)	1.05 (0.76-1.46)	0%	
	Ethnicity	Ashkenazi	5	1.85 (1.16-2.97)	1.79 (0.98-3.25)	29%	0.9
		Non-Ashkenazi European ancestry	12	1.49 (1.20-1.84)	1.63 (1.15-2.31)	45%	
		African ancestry	2	1.82 (0.32-10.23)	1.82 (0.32-10.23)	0%	
		Asian ancestry	1	2.27 (0.92-5.59)	2.27 (0.92-5.59)	–	
	Age adjustment	No adjustment for age	5	2.22 (1.50-3.30)	2.42 (1.31-4.48)	57%	0.11
		Age differences adjusted for or addressed through study design, or age distribution was similar between the comparison groups	15	1.42 (1.14-1.76)	1.42 (1.14-1.78)	1%	
	Case/participant selection	Selected for young age at prostate cancer onset	1	3.75 (1.22-11.50)	3.75 (1.22-11.50)	–	0.056
		Selected for aggressive disease	3	3.52 (1.69-7.31)	3.52 (1.69-7.31)	0%	
		Selected for family history of prostate cancer	1	4.00 (0.48-33.13)	4.00 (0.48-33.13)	–	
		Unselected for age, aggressive prostate cancer and family history	15	1.43 (1.17-1.75)	1.47 (1.13-1.91)	25%	
	Control selection	Non-prostate cancer patients	3	2.12 (1.05-4.30)	2.12 (1.05-4.30)	0%	0.5
		All other studies	17	1.54 (1.26-1.87)	1.66 (1.23-2.23)	39%	
	Control selection (Case-control studies only)	Non-prostate cancer patients	3	2.12 (1.05-4.30)	2.12 (1.05-4.30)	0%	0.8
		Population controls	12	1.93 (1.39-2.68)	1.96 (1.28-3.01)	32%	
	Historical or external controls	Historical controls or external population estimates	4	3.14 (1.97-4.99)	3.14 (1.97-4.99)	0%	0.002
		All other studies	16	1.37 (1.11-1.69)	1.38 (1.11-1.72)	3%	
	Historical or external controls (Case-control studies only)	Historical controls or external population estimates	4	3.14 (1.97-4.99)	3.14 (1.97-4.99)	0%	0.010
		Controls from the same population as the cases	11	1.42 (0.97-2.10)	1.42 (0.97-2.10)	0%	

Continued on next page

Table S2: (continued)

Gene	Covariate	Subgroup	No. studies	Fixed effects pooled RR (95% CI)	Random effects pooled RR (95% CI)	I^2	p-value[a]
BRCA2	Overall	All estimates	21	5.24 (4.63-5.94)	3.94 (2.79-5.56)	83%	
	Study design	Case-control	15	7.00 (5.82-8.42)	4.00 (2.38-6.73)	83%	0.8
		Prospective cohort	2	3.47 (2.49-4.84)	3.04 (1.36-6.82)	80%	
		Family-based retrospective cohort	4	4.35 (3.57-5.30)	4.00 (2.61-6.13)	74%	
	Ethnicity	Ashkenazi	6	2.08 (1.38-3.12)	2.08 (1.38-3.12)	0%	0.006
		Non-Ashkenazi European ancestry	12	5.82 (5.08-6.68)	4.97 (3.24-7.62)	88%	
		African ancestry	2	3.19 (1.01-10.09)	3.67 (0.73-18.38)	43%	
		Asian ancestry	1	5.65 (3.49-9.15)	5.65 (3.49-9.15)	–	
	Age adjustment	No adjustment for age	7	10.32 (8.17-13.04)	4.85 (2.18-10.79)	86%	0.5
		Age differences adjusted for or addressed through study design, or age distribution was similar between the comparison groups	14	4.00 (3.45-4.64)	3.63 (2.84-4.63)	51%	
	Case/participant selection	Selected for young age at prostate cancer onset	3	8.34 (5.64-12.31)	8.34 (5.64-12.31)	0%	<0.001
		Selected for aggressive disease	3	15.10 (11.11-20.51)	5.77 (1.25-26.63)	87%	
		Selected for family history of prostate cancer	0	–	–	–	
		Unselected for age, aggressive prostate cancer and family history	15	3.87 (3.34-4.47)	3.33 (2.57-4.33)	58%	
	Control selection	Non-prostate cancer patients	2	5.83 (3.64-9.32)	5.83 (3.64-9.32)	0%	0.15
		All other studies	19	5.20 (4.57-5.92)	3.74 (2.57-5.43)	84%	
	Control selection (Case-control studies only)	Non-prostate cancer patients	2	5.83 (3.64-9.32)	5.83 (3.64-9.32)	0%	0.2
		Population controls	13	7.24 (5.92-8.84)	3.63 (1.98-6.65)	85%	
	Historical or external controls	Historical controls or external population estimates	6	10.37 (8.20-13.13)	4.69 (2.00-10.98)	89%	0.6
		All other studies	15	4.01 (3.46-4.65)	3.66 (2.89-4.65)	48%	
	Historical or external controls (Case-control studies only)	Historical controls or external population estimates	6	10.37 (8.20-13.13)	4.69 (2.00-10.98)	89%	0.6
		Controls from the same population as the cases	9	3.75 (2.79-5.05)	3.63 (2.60-5.07)	11%	

Continued on next page

Table S2: (continued)

Gene	Covariate	Subgroup	No. studies	Fixed effects pooled RR (95% CI)	Random effects pooled RR (95% CI)	I^2	p-value[a]
	Proportion of PVs located in OCCR	≥50% OCCR	11	6.19 (5.13-7.47)	3.41 (1.75-6.61)	90%	0.8
		<50% OCCR	6	4.73 (3.82-5.87)	4.43 (3.06-6.41)	56%	
		Not determinable	4	4.41 (3.38-5.74)	4.41 (3.38-5.74)	0%	

[a] Test for subgroup differences.

Table S3: Heterogeneity and pooled RR estimates by study subgroups, after restriction to studies of participants unselected for age at diagnosis, prostate cancer family history, and disease aggressiveness.
Abbreviations: RR, relative risk. CI, confidence interval. PV, pathogenic variant. OCCR, ovarian cancer cluster region.

Gene	Covariate	Subgroup	No. studies	Fixed effects pooled RR (95% CI)	Random effects pooled RR (95% CI)	I^2	p-value[a]
BRCA1	Overall	All estimates	15	1.43 (1.17-1.75)	1.47 (1.13-1.91)	25%	
	Study design	Case-control	10	1.60 (1.13-2.25)	1.59 (1.07-2.36)	17%	0.13
		Prospective cohort	2	1.87 (1.28-2.74)	1.83 (1.07-3.12)	48%	
		Family-based retrospective cohort	3	1.05 (0.76-1.46)	1.05 (0.76-1.46)	0%	
	Ethnicity	Ashkenazi	5	1.85 (1.16-2.97)	1.79 (0.98-3.25)	29%	0.6
		Non-Ashkenazi European ancestry	8	1.30 (1.03-1.64)	1.30 (0.95-1.79)	30%	
		African ancestry	1	1.11 (0.09-13.61)	1.11 (0.09-13.61)	–	
		Asian ancestry	1	2.27 (0.92-5.59)	2.27 (0.92-5.59)	–	
	Age adjustment	No adjustment for age	3	1.72 (1.07-2.78)	1.88 (0.81-4.34)	66%	0.5
		Age differences adjusted for or addressed through study design, or age distribution was similar between the comparison groups	12	1.37 (1.10-1.71)	1.38 (1.08-1.77)	8%	
	Control selection	Non-prostate cancer patients	3	2.12 (1.05-4.30)	2.12 (1.05-4.30)	0%	0.3
		All other studies	12	1.38 (1.12-1.70)	1.40 (1.03-1.89)	35%	
	Control selection (Case-control studies only)	Non-prostate cancer patients	3	2.12 (1.05-4.30)	2.12 (1.05-4.30)	0%	0.4
		Population controls	7	1.46 (0.98-2.17)	1.41 (0.81-2.44)	38%	
	Historical or external controls	Historical controls or external population estimates	2	2.68 (1.44-4.97)	2.70 (1.41-5.14)	7%	0.044
		All other studies	13	1.32 (1.07-1.64)	1.33 (1.05-1.69)	8%	
	Historical or external controls (Case-control studies only)	Historical controls or external population estimates	2	2.68 (1.44-4.97)	2.70 (1.41-5.14)	7%	0.053
		Controls from the same population as the cases	8	1.27 (0.84-1.92)	1.27 (0.84-1.92)	0%	
BRCA2	Overall	All estimates	15	3.87 (3.34-4.47)	3.33 (2.57-4.33)	58%	

Continued on next page

Table S3: (continued)

**Gene**	**Covariate**	**Subgroup**	**No. studies**	**Fixed effects pooled RR (95% CI)**	**Random effects pooled RR (95% CI)**	I^2	**p-value[a]**
	Study design	Case-control	9	3.27 (2.46-4.35)	2.86 (1.87-4.37)	42%	0.5
		Prospective cohort	2	3.47 (2.49-4.84)	3.04 (1.36-6.82)	80%	
		Family-based retrospective cohort	4	4.35 (3.57-5.30)	4.00 (2.61-6.13)	74%	
	Ethnicity	Ashkenazi	6	2.08 (1.38-3.12)	2.08 (1.38-3.12)	0%	0.011
		Non-Ashkenazi European ancestry	7	4.07 (3.45-4.80)	3.69 (2.71-5.04)	66%	
		African ancestry	1	10.30 (1.28-82.73)	10.30 (1.28-82.73)	–	
		Asian ancestry	1	5.65 (3.49-9.15)	5.65 (3.49-9.15)	–	
	Age adjustment	No adjustment for age	3	1.63 (0.84-3.17)	1.63 (0.84-3.17)	0%	0.026
		Age differences adjusted for or addressed through study design, or age distribution was similar between the comparison groups	12	4.04 (3.48-4.69)	3.67 (2.83-4.76)	57%	
	Control selection	Non-prostate cancer patients	2	5.83 (3.64-9.32)	5.83 (3.64-9.32)	0%	0.023
		All other studies	13	3.70 (3.18-4.32)	3.08 (2.33-4.08)	60%	
	Control selection (Case-control studies only)	Non-prostate cancer patients	2	5.83 (3.64-9.32)	5.83 (3.64-9.32)	0%	0.002
		Population controls	7	2.33 (1.63-3.34)	2.33 (1.63-3.34)	0%	
	Historical or external controls	Historical controls or external population estimates	3	1.63 (0.84-3.17)	1.63 (0.84-3.17)	0%	0.026
		All other studies	12	4.04 (3.48-4.69)	3.67 (2.83-4.76)	57%	
	Historical or external controls (Case-control studies only)	Historical controls or external population estimates	3	1.63 (0.84-3.17)	1.63 (0.84-3.17)	0%	0.054
		Controls from the same population as the cases	6	3.83 (2.79-5.25)	3.58 (2.29-5.57)	36%	
	Proportion of PVs located in OCCR	≥50% OCCR	8	2.30 (1.74-3.06)	2.30 (1.74-3.06)	0%	0.002
		<50% OCCR	4	4.74 (3.81-5.91)	4.38 (2.83-6.77)	73%	
		Not determinable	3	4.55 (3.48-5.95)	4.55 (3.48-5.95)	0%	

[a] Test for subgroup differences.

Table S4: Heterogeneity and pooled RR estimates by study subgroups, restricted to *BRCA1* studies that did not use historical controls.
Abbreviations: RR, relative risk. CI, confidence interval.

**Gene, subgroup**	**Covariate**	**Subgroup**	**No. studies**	**Fixed effects pooled RR (95% CI)**	**Random effects pooled RR (95% CI)**	I^2	**p-value**[a]
BRCA1, no historical controls	Overall	All estimates	13	1.32 (1.07-1.64)	1.33 (1.05-1.69)	8%	
	Study design	Case-control	8	1.27 (0.84-1.92)	1.27 (0.84-1.92)	0%	0.2
		Prospective cohort	2	1.87 (1.28-2.74)	1.83 (1.07-3.12)	48%	
		Family-based retrospective cohort	3	1.05 (0.76-1.46)	1.05 (0.76-1.46)	0%	
	Ethnicity	Ashkenazi	3	1.12 (0.55-2.31)	1.12 (0.55-2.31)	0%	0.7
		Non-Ashkenazi European ancestry	8	1.30 (1.03-1.64)	1.30 (0.95-1.79)	30%	
		African ancestry	1	1.11 (0.09-13.61)	1.11 (0.09-13.61)	–	
		Asian ancestry	1	2.27 (0.92-5.59)	2.27 (0.92-5.59)	–	
	Age adjustment	No adjustment for age	1	0.90 (0.42-1.91)	0.90 (0.42-1.91)	–	0.3
		Age differences adjusted for or addressed through study design, or age distribution was similar between the comparison groups	12	1.37 (1.10-1.71)	1.38 (1.08-1.77)	8%	
	Control selection	Non-prostate cancer patients	3	2.12 (1.05-4.30)	2.12 (1.05-4.30)	0%	0.17
		All other studies	10	1.26 (1.01-1.58)	1.25 (0.95-1.65)	17%	
	Control selection (Case-control studies only)	Cases vs controls with non-prostate cancer	3	2.12 (1.05-4.30)	2.12 (1.05-4.30)	0%	0.076
		Cases vs unselected controls	5	0.97 (0.58-1.61)	0.97 (0.58-1.61)	0%	

[a] Test for subgroup differences.

Table S5: Leave-one-out analysis of *BRCA1* studies, after restriction to studies that did not use historical controls or external population frequency estimates

**Publication**	I^2	**p-value**	**Fixed effects RR (95% CI)**	**Random effects RR (95% CI)**
All studies	8%	–	1.32 (1.07-1.64)	1.33 (1.05-1.69)
Excluding: Hubert 1999	15%	0.8	1.33 (1.07-1.65)	1.34 (1.04-1.73)
Excluding: Thompson 2002	0%	0.15	1.49 (1.14-1.94)	1.49 (1.14-1.94)
Excluding: Risch 2006	14%	0.6	1.33 (1.07-1.65)	1.34 (1.04-1.72)
Excluding: Agalliu 2009	16%	0.9	1.32 (1.06-1.65)	1.33 (1.01-1.74)
Excluding: Gallagher 2010	5%	0.2	1.34 (1.08-1.67)	1.35 (1.08-1.70)
Excluding: Fachal 2011	6%	0.3	1.34 (1.08-1.66)	1.35 (1.07-1.70)
Excluding: Moran 2012	13%	0.5	1.35 (1.08-1.68)	1.36 (1.05-1.77)
Excluding: Cybulski 2013	8%	0.3	1.37 (1.10-1.71)	1.38 (1.08-1.77)
Excluding: Page 2019	16%	0.9	1.32 (1.05-1.66)	1.33 (1.00-1.76)
Excluding: Matejcic 2020, Uganda	16%	0.9	1.33 (1.07-1.64)	1.33 (1.03-1.72)
Excluding: Momozawa 2020	5%	0.2	1.28 (1.03-1.60)	1.28 (1.01-1.63)
Excluding: Nyberg 2020	0%	0.013	1.16 (0.92-1.47)	1.16 (0.92-1.47)
Excluding: Oak 2020	11%	0.4	1.30 (1.05-1.62)	1.31 (1.02-1.68)

Table S6: Heterogeneity and pooled RR estimates by study subgroups, for *BRCA2* studies by ethnicity. Abbreviations: RR, relative risk. CI, confidence interval. PV, pathogenic variant. OCCR, ovarian cancer cluster region.

**Gene, subgroup**	**Covariate**	**Subgroup**	**No. studies**	**Fixed effects pooled RR (95% CI)**	**Random effects pooled RR (95% CI)**	I^2	**p-value[a]**
BRCA2, Ashkenazi ancestry	Overall	All estimates	6	2.08 (1.38-3.12)	2.08 (1.38-3.12)	0%	
	Study design	Case-control	6	2.08 (1.38-3.12)	2.08 (1.38-3.12)	0%	–
		Prospective cohort	0	–	–	–	
		Family-based retrospective cohort	0	–	–	–	
	Age adjustment	No adjustment for age	3	1.63 (0.84-3.17)	1.63 (0.84-3.17)	0%	0.4
		Age differences adjusted for or addressed through study design, or age distribution was similar between the comparison groups	3	2.40 (1.43-4.03)	2.40 (1.43-4.03)	0%	
	Control selection	Non-prostate cancer patients	0	–	–	–	–
		All other studies	6	2.08 (1.38-3.12)	2.08 (1.38-3.12)	0%	
	Historical or external controls	Historical controls or external population estimates	3	1.63 (0.84-3.17)	1.63 (0.84-3.17)	0%	0.4
		All other studies	3	2.40 (1.43-4.03)	2.40 (1.43-4.03)	0%	
	Historical or external controls (Case-control studies only)	Historical controls or external population estimates	3	1.63 (0.84-3.17)	1.63 (0.84-3.17)	0%	0.4
		Controls from the same population as the cases	3	2.40 (1.43-4.03)	2.40 (1.43-4.03)	0%	
	Proportion of PVs located in OCCR	≥50% OCCR	6	2.08 (1.38-3.12)	2.08 (1.38-3.12)	0%	–
		<50% OCCR	0	–	–	–	
		Not determinable	0	–	–	–	
BRCA2, non-Ashkenazi European ancestry	Overall	All estimates	7	4.07 (3.45-4.80)	3.69 (2.71-5.04)	66%	
	Study design	Case-control	1	3.50 (1.63-7.50)	3.50 (1.63-7.50)	–	0.8
		Prospective cohort	2	3.47 (2.49-4.84)	3.04 (1.36-6.82)	80%	
		Family-based retrospective cohort	4	4.35 (3.57-5.30)	4.00 (2.61-6.13)	74%	
	Age adjustment	No adjustment for age	0	–	–	–	–
		Age differences adjusted for or addressed through study design, or age distribution was similar between the comparison groups	7	4.07 (3.45-4.80)	3.69 (2.71-5.04)	66%	–
	Control selection	Non-prostate cancer patients	0	–	–	–	–

Continued on next page

Table S6: (continued)

**Gene, subgroup**	**Covariate**	**Subgroup**	**No. studies**	**Fixed effects pooled RR (95% CI)**	**Random effects pooled RR (95% CI)**	I^2	**p-value**[a]
		All other studies	7	4.07 (3.45-4.80)	3.69 (2.71-5.04)	66%	–
	Historical or external controls	Historical controls or external population estimates	0	–	–	–	–
		All other studies	7	4.07 (3.45-4.80)	3.69 (2.71-5.04)	66%	–
	Proportion of PVs located in OCCR	≥50% OCCR	2	2.53 (1.71-3.75)	2.53 (1.71-3.75)	0%	0.063
		<50% OCCR	3	4.53 (3.54-5.80)	3.97 (2.20-7.16)	81%	
		Not determinable	2	4.49 (3.42-5.88)	4.49 (3.42-5.88)	0%	
BRCA2, African ancestry	Overall	All estimates	1	10.30 (1.28-82.73)	10.30 (1.28-82.73)	–	
BRCA2, Asian ancestry	Overall	All estimates	1	5.65 (3.49-9.15)	5.65 (3.49-9.15)	–	

[a] Test for subgroup differences.

Table S7: Leave-one-out analysis of *BRCA2* studies, by ethnicity.

**Population**	**Publication**	I^2	**p-value**	**Fixed effects RR (95% CI)**	**Random effects RR (95% CI)**
Ashkenazi ancestry	All studies	0%	–	2.08 (1.38-3.12)	2.08 (1.38-3.12)
	Excluding: Hubert 1999	0%	0.6	2.11 (1.40-3.19)	2.11 (1.40-3.19)
	Excluding: Vazina 2000	0%	0.5	2.14 (1.41-3.25)	2.14 (1.41-3.25)
	Excluding: Giusti 2003	0%	0.9	2.10 (1.31-3.36)	2.10 (1.31-3.36)
	Excluding: Hamel 2003	0%	0.4	2.20 (1.44-3.37)	2.20 (1.44-3.37)
	Excluding: Agalliu 2009	0%	0.8	2.15 (1.32-3.49)	2.15 (1.32-3.49)
	Excluding: Gallagher 2010	0%	0.18	1.72 (1.05-2.81)	1.72 (1.05-2.81)
Non-Ashkenazi European ancestry	All studies	66%	–	4.07 (3.45-4.80)	3.69 (2.71-5.04)
	Excluding: BCLC 1999	70%	0.3	3.82 (3.12-4.67)	3.45 (2.33-5.11)
	Excluding: van Asperen 2005	59%	0.017	4.43 (3.70-5.29)	4.03 (2.96-5.50)
	Excluding: Risch 2006	71%	0.4	4.13 (3.49-4.88)	3.78 (2.72-5.26)
	Excluding: Moran 2012	55%	0.010	3.65 (3.03-4.39)	3.33 (2.45-4.53)
	Excluding: Akbari 2014	72%	0.7	4.10 (3.46-4.86)	3.69 (2.62-5.20)
	Excluding: Page 2019	57%	0.013	4.32 (3.64-5.13)	4.09 (3.07-5.45)
	Excluding: Nyberg 2020	72%	0.6	4.00 (3.33-4.79)	3.51 (2.40-5.13)

Table S8: Heterogeneity and pooled RR estimates by study subgroups, for *BRCA2* studies by the proportion of pathogenic variants in the ovarian cancer cluster region.
Abbreviations: RR, relative risk. CI, confidence interval. PV, pathogenic variant. OCCR, ovarian cancer cluster region.

**Gene, subgroup**	**Covariate**	**Subgroup**	**No. studies**	**Fixed effects pooled RR (95% CI)**	**Random effects pooled RR (95% CI)**	I^2	**p-value**[a]
BRCA2, ≥50% OCCR PVs	Overall	All estimates	8	2.30 (1.74-3.06)	2.30 (1.74-3.06)	0%	
	Study design	Case-control	6	2.08 (1.38-3.12)	2.08 (1.38-3.12)	0%	0.5
		Prospective cohort	0	–	–	–	
		Family-based retrospective cohort	2	2.53 (1.71-3.75)	2.53 (1.71-3.75)	0%	
	Ethnicity	Ashkenazi	6	2.08 (1.38-3.12)	2.08 (1.38-3.12)	0%	0.5
		Non-Ashkenazi European ancestry	2	2.53 (1.71-3.75)	2.53 (1.71-3.75)	0%	
		African ancestry	0	–	–	–	
		Asian ancestry	0	–	–	–	
	Age adjustment	No adjustment for age	3	1.63 (0.84-3.17)	1.63 (0.84-3.17)	0%	0.3
		Age differences adjusted for or addressed through study design, or age distribution was similar between the comparison groups	5	2.49 (1.82-3.40)	2.49 (1.82-3.40)	0%	
	Control selection	Non-prostate cancer patients	0	–	–	–	–
		All other studies	8	2.30 (1.74-3.06)	2.30 (1.74-3.06)	0%	
	Historical or external controls	Historical controls or external population estimates	3	1.63 (0.84-3.17)	1.63 (0.84-3.17)	0%	0.3
		All other studies	5	2.49 (1.82-3.40)	2.49 (1.82-3.40)	0%	
	Historical or external controls (Case-control studies only)	Historical controls or external population estimates	3	1.63 (0.84-3.17)	1.63 (0.84-3.17)	0%	0.4
		Controls from the same population as the cases	3	2.40 (1.43-4.03)	2.40 (1.43-4.03)	0%	
BRCA2, <50% OCCR PVs	Overall	All estimates	4	4.74 (3.81-5.91)	4.38 (2.83-6.77)	73%	
	Study design	Case-control	1	5.65 (3.49-9.15)	5.65 (3.49-9.15)	–	0.3
		Prospective cohort	2	3.47 (2.49-4.84)	3.04 (1.36-6.82)	80%	
		Family-based retrospective cohort	1	6.30 (4.35-9.11)	6.30 (4.35-9.11)	–	

Continued on next page

Table S8: (continued)

Gene, subgroup	Covariate	Subgroup	No. studies	Fixed effects pooled RR (95% CI)	Random effects pooled RR (95% CI)	I^2	p-value[a]
	Ethnicity	Ashkenazi	0	–	–	–	0.4
		Non-Ashkenazi European ancestry	3	4.53 (3.54-5.80)	3.97 (2.20-7.16)	81%	
		African ancestry	0	–	–	–	
		Asian ancestry	1	5.65 (3.49-9.15)	5.65 (3.49-9.15)	–	
	Age adjustment	No adjustment for age	0	–	–	–	–
		Age differences adjusted for or addressed through study design, or age distribution was similar between the comparison groups	4	4.74 (3.81-5.91)	4.38 (2.83-6.77)	73%	
	Control selection	Non-prostate cancer patients	1	5.65 (3.49-9.15)	5.65 (3.49-9.15)	–	0.4
		All other studies	3	4.53 (3.54-5.80)	3.97 (2.20-7.16)	81%	
	Control selection (Case-control studies only)	Non-prostate cancer patients	1	5.65 (3.49-9.15)	5.65 (3.49-9.15)	–	
		Population controls	0	–	–	–	
	Historical or external controls	Historical controls or external population estimates	0	–	–	–	–
		All other studies	4	4.74 (3.81-5.91)	4.38 (2.83-6.77)	73%	
BRCA2, Proportion OCCR PVs not determinable	Overall	All estimates	3	4.55 (3.48-5.95)	4.55 (3.48-5.95)	0%	
	Study design	Case-control	2	3.98 (1.94-8.13)	3.98 (1.94-8.13)	0%	0.7
		Prospective cohort	0	–	–	–	
		Family-based retrospective cohort	1	4.65 (3.48-6.22)	4.65 (3.48-6.22)	–	
	Ethnicity	Ashkenazi	0	–	–	–	0.4
		Non-Ashkenazi European ancestry	2	4.49 (3.42-5.88)	4.49 (3.42-5.88)	0%	
		African ancestry	1	10.30 (1.28-82.73)	10.30 (1.28-82.73)	–	
		Asian ancestry	0	–	–	–	
	Age adjustment	No adjustment for age	0	–	–	–	–
		Age differences adjusted for or addressed through study design, or age distribution was similar between the comparison groups	3	4.55 (3.48-5.95)	4.55 (3.48-5.95)	0%	
	Control selection	Non-prostate cancer patients	1	10.30 (1.28-82.73)	10.30 (1.28-82.73)	–	0.4
		All other studies	2	4.49 (3.42-5.88)	4.49 (3.42-5.88)	0%	

Continued on next page

Table S8: (continued)

**Gene, subgroup**	**Covariate**	**Subgroup**	**No. studies**	**Fixed effects pooled RR (95% CI)**	**Random effects pooled RR (95% CI)**	I^2	**p-value**[a]
	Control selection (Case-control studies only)	Non-prostate cancer patients	1	10.30 (1.28-82.73)	10.30 (1.28-82.73)	–	0.3
		Population controls	1	3.50 (1.63-7.50)	3.50 (1.63-7.50)	–	
	Historical or external controls	Historical controls or external population estimates	0	–	–	–	–
		All other studies	3	4.55 (3.48-5.95)	4.55 (3.48-5.95)	0%	

[a] Test for subgroup differences.

Table S9: Leave-one-out analysis of *BRCA2* studies, by proportion of considered pathogenic variants that were located in the ovarian cancer cluster region.
Abbreviations: RR, relative risk. CI, confidence interval. PV, pathogenic variant. OCCR, ovarian cancer cluster region.

**Proportion PVs in OCCR**	**Publication**	I^2	**p-value**	**Fixed effects RR (95% CI)**	**Random effects RR (95% CI)**
≥50% OCCR PVs	All studies	0%	–	2.30 (1.74-3.06)	2.30 (1.74-3.06)
	Excluding: Hubert 1999	0%	0.6	2.32 (1.75-3.09)	2.32 (1.75-3.09)
	Excluding: Vazina 2000	0%	0.4	2.34 (1.76-3.12)	2.34 (1.76-3.12)
	Excluding: Giusti 2003	0%	0.7	2.34 (1.73-3.17)	2.34 (1.73-3.17)
	Excluding: Hamel 2003	0%	0.3	2.38 (1.78-3.17)	2.38 (1.78-3.17)
	Excluding: van Asperen 2005	0%	0.6	2.17 (1.49-3.15)	2.17 (1.49-3.15)
	Excluding: Risch 2006	0%	0.7	2.27 (1.68-3.05)	2.27 (1.68-3.05)
	Excluding: Agalliu 2009	0%	0.6	2.37 (1.75-3.22)	2.37 (1.75-3.22)
	Excluding: Gallagher 2010	0%	0.4	2.18 (1.60-2.96)	2.18 (1.60-2.96)
<50% OCCR PVs	All studies	73%	–	4.74 (3.81-5.91)	4.38 (2.83-6.77)
	Excluding: Moran 2012	74%	0.061	4.06 (3.09-5.34)	3.78 (2.18-6.57)
	Excluding: Page 2019	0%	0.002	5.43 (4.29-6.87)	5.43 (4.29-6.87)
	Excluding: Momozawa 2020	81%	0.4	4.53 (3.54-5.80)	3.97 (2.20-7.16)
	Excluding: Nyberg 2020	82%	0.7	4.88 (3.75-6.35)	4.26 (2.23-8.15)
Proportion OCCR PVs not determinable	All studies	0%	–	4.55 (3.48-5.95)	4.55 (3.48-5.95)
	Excluding: BCLC 1999	0%	0.7	3.98 (1.94-8.13)	3.98 (1.94-8.13)
	Excluding: Akbari 2014	0%	0.5	4.72 (3.54-6.29)	4.72 (3.54-6.29)
	Excluding: Matejcic 2020, Uganda	0%	0.4	4.49 (3.42-5.88)	4.49 (3.42-5.88)

Table S10: Heterogeneity and pooled RR estimates by study subgroups, for *BRCA1* carriers younger or older than 65 years.
Abbreviations: RR, relative risk. CI, confidence interval.

**Gene, age group**	**Covariate**	**Subgroup**	**No. studies**	**Fixed effects pooled RR (95% CI)**	**Random effects pooled RR (95% CI)**	I^2	**p-value**[a]
BRCA1, age<65	Overall	All estimates	4	2.21 (1.47-3.30)	2.19 (1.21-3.98)	47%	
	Study design	Case-control	2	1.81 (0.80-4.09)	1.75 (0.38-8.03)	71%	0.4
		Prospective cohort	1	3.57 (1.68-7.58)	3.57 (1.68-7.58)	–	
		Family-based retrospective cohort	1	1.82 (1.01-3.28)	1.82 (1.01-3.28)	–	
	Ethnicity	Ashkenazi	1	0.79 (0.24-2.61)	0.79 (0.24-2.61)	–	0.071
		Non-Ashkenazi European ancestry	3	2.52 (1.64-3.87)	2.59 (1.58-4.24)	19%	
		African ancestry	0	–	–	–	
		Asian ancestry	0	–	–	–	
	Control selection	Non-prostate cancer patients	0	–	–	–	–
		All other studies	4	2.21 (1.47-3.30)	2.19 (1.21-3.98)	47%	
	Historical or external controls	Historical controls or external population estimates	1	3.75 (1.22-11.50)	3.75 (1.22-11.50)	–	0.3
		All other studies	3	2.04 (1.32-3.14)	1.92 (0.94-3.92)	57%	
	Historical or external controls (Case-control studies only)	Historical controls or external population estimates	1	3.75 (1.22-11.50)	3.75 (1.22-11.50)	–	0.062
		Controls from the same population as the cases	1	0.79 (0.24-2.61)	0.79 (0.24-2.61)	–	
BRCA1, age≥65	Overall	All estimates	3	1.18 (0.83-1.70)	1.43 (0.71-2.87)	65%	
	Study design	Case-control	1	2.70 (0.82-8.88)	2.70 (0.82-8.88)	–	0.056
		Prospective cohort	1	1.86 (0.96-3.60)	1.86 (0.96-3.60)	–	
		Family-based retrospective cohort	1	0.84 (0.53-1.33)	0.84 (0.53-1.33)	–	
	Ethnicity	Ashkenazi	1	2.70 (0.82-8.88)	2.70 (0.82-8.88)	–	0.3
		Non-Ashkenazi European ancestry	2	1.09 (0.75-1.59)	1.21 (0.55-2.62)	73%	
		African ancestry	0	–	–	–	
		Asian ancestry	0	–	–	–	
	Control selection	Non-prostate cancer patients	0	–	–	–	–
		All other studies	3	1.18 (0.83-1.70)	1.43 (0.71-2.87)	65%	

Continued on next page

Table S10: (continued)

**Gene, age group**	**Covariate**	**Subgroup**	**No. studies**	**Fixed effects pooled RR (95% CI)**	**Random effects pooled RR (95% CI)**	I^2	**p-value**[a]
	Historical or external controls	Historical controls or external population estimates	0	–	–	–	–
		All other studies	3	1.18 (0.83-1.70)	1.43 (0.71-2.87)	65%	

[a] Test for subgroup differences.

Table S11: Leave-one-out analysis of *BRCA1* RR estimates at ages younger or older than 65 years.

**Age group**	**Publication**	I^2	**p-value**	**Fixed effects RR (95% CI)**	**Random effects RR (95% CI)**
Age <65	All studies	47%	–	2.21 (1.47-3.30)	2.19 (1.21-3.98)
	Excluding: Thompson 2002	59%	0.4	2.61 (1.50-4.54)	2.35 (0.94-5.86)
	Excluding: Agalliu 2009	19%	0.073	2.52 (1.64-3.87)	2.59 (1.58-4.24)
	Excluding: Leongamornlert 2012	57%	0.3	2.04 (1.32-3.14)	1.92 (0.94-3.92)
	Excluding: Nyberg 2020	43%	0.14	1.82 (1.13-2.93)	1.80 (0.88-3.66)
Age ≥65	All studies	65%	–	1.18 (0.83-1.70)	1.43 (0.71-2.87)
	Excluding: Thompson 2002	0%	0.019	2.03 (1.14-3.61)	2.03 (1.14-3.61)
	Excluding: Agalliu 2009	73%	0.15	1.09 (0.75-1.59)	1.21 (0.55-2.62)
	Excluding: Nyberg 2020	69%	0.11	0.98 (0.64-1.50)	1.32 (0.43-4.01)

Table S12: Heterogeneity and pooled RR estimates by study subgroups, for *BRCA2* carriers younger or older than 65 years.
Abbreviations: RR, relative risk. CI, confidence interval. PV, pathogenic variant. OCCR, ovarian cancer cluster region.

Gene, age group	Covariate	Subgroup	No. studies	Fixed effects pooled RR (95% CI)	Random effects pooled RR (95% CI)	I^2	p-value[a]
BRCA2, age<65	Overall	All estimates	5	6.37 (4.81-8.43)	5.28 (3.10-9.00)	63%	
	Study design	Case-control	3	6.52 (4.34-9.78)	4.44 (1.25-15.84)	77%	0.4
		Prospective cohort	1	3.99 (1.88-8.48)	3.99 (1.88-8.48)	–	
		Family-based retrospective cohort	1	7.33 (4.66-11.52)	7.33 (4.66-11.52)	–	
	Ethnicity	Ashkenazi	1	1.58 (0.57-4.38)	1.58 (0.57-4.38)	–	0.005
		Non-Ashkenazi European ancestry	4	7.14 (5.33-9.56)	7.14 (5.33-9.56)	0%	
		African ancestry	0	–	–	–	
		Asian ancestry	0	–	–	–	
	Control selection	Non-prostate cancer patients	0	–	–	0%	–
		All other studies	5	6.37 (4.81-8.43)	5.28 (3.10-9.00)	63%	
	Historical or external controls	Historical controls or external population estimates	1	8.60 (5.47-13.52)	8.60 (5.47-13.52)	–	0.10
		All other studies	4	5.28 (3.69-7.55)	4.23 (2.06-8.68)	62%	
	Historical or external controls (Case-control studies only)	Historical controls or external population estimates	1	8.60 (5.47-13.52)	8.60 (5.47-13.52)	–	0.057
		Controls from the same population as the cases	2	2.07 (0.82-5.18)	2.35 (0.67-8.26)	29%	
	Proportion of PVs located in OCCR	≥50% OCCR	2	6.51 (4.31-9.85)	3.93 (0.75-20.58)	89%	0.4
		<50% OCCR	2	4.22 (2.07-8.59)	4.22 (2.07-8.59)	0%	
		Not determinable	1	7.33 (4.66-11.52)	7.33 (4.66-11.52)	–	
BRCA2, age≥65	Overall	All estimates	3	3.74 (2.82-4.96)	3.74 (2.82-4.96)	0%	
	Study design	Case-control	1	2.63 (0.85-8.16)	2.63 (0.85-8.16)	–	0.5
		Prospective cohort	1	4.64 (2.91-7.40)	4.64 (2.91-7.40)	–	
		Family-based retrospective cohort	1	3.39 (2.34-4.92)	3.39 (2.34-4.92)	–	

Continued on next page

Table S12: (continued)

**Gene, age group**	**Covariate**	**Subgroup**	**No. studies**	**Fixed effects pooled RR (95% CI)**	**Random effects pooled RR (95% CI)**	I^2	**p-value**[a]
	Ethnicity	Ashkenazi	1	2.63 (0.85-8.16)	2.63 (0.85-8.16)	–	0.5
		Non-Ashkenazi European ancestry	2	3.83 (2.86-5.12)	3.84 (2.84-5.18)	6%	
		African ancestry	0	–	–	–	
		Asian ancestry	0	–	–	–	
	Control selection	Non-prostate cancer patients	0	–	–	–	–
		All other studies	3	3.74 (2.82-4.96)	3.74 (2.82-4.96)	0%	
	Historical or external controls	Historical controls or external population estimates	0	–	–	–	–
		All other studies	3	3.74 (2.82-4.96)	3.74 (2.82-4.96)	0%	
	Proportion of PVs located in OCCR	≥50% OCCR	1	2.63 (0.85-8.16)	2.63 (0.85-8.16)	–	0.5
		<50% OCCR	1	4.64 (2.91-7.40)	4.64 (2.91-7.40)	–	
		Not determinable	1	3.39 (2.34-4.92)	3.39 (2.34-4.92)	–	

[a] Test for subgroup differences.

Table S13: Leave-one-out analysis of *BRCA2* RR estimates at ages younger or older than 65 years.

**Age group**	**Publication**	I^2	**p-value**	**Fixed effects RR (95% CI)**	**Random effects RR (95% CI)**
Age <65	All studies	63%	–	6.37 (4.81-8.43)	5.28 (3.10-9.00)
	Excluding: Johannesdottir 1996	72%	0.97	6.37 (4.80-8.45)	5.14 (2.87-9.20)
	Excluding: BCLC 1999	70%	0.4	5.84 (4.08-8.34)	4.40 (1.95-9.94)
	Excluding: Agalliu 2009	0%	0.005	7.14 (5.33-9.56)	7.14 (5.33-9.56)
	Excluding: Kote-Jarai 2011	62%	0.097	5.28 (3.69-7.55)	4.23 (2.06-8.68)
	Excluding: Nyberg 2020	67%	0.19	6.87 (5.08-9.29)	5.61 (2.97-10.61)
Age $\geq$65	All studies	0%	–	3.74 (2.82-4.96)	3.74 (2.82-4.96)
	Excluding: BCLC 1999	0%	0.4	4.27 (2.77-6.58)	4.27 (2.77-6.58)
	Excluding: Agalliu 2009	6%	0.5	3.83 (2.86-5.12)	3.84 (2.84-5.18)
	Excluding: Nyberg 2020	0%	0.3	3.31 (2.32-4.71)	3.31 (2.32-4.71)

Figure S1: Funnel plots of overall *BRCA1* RR estimates.

(a) all initially considered studies (test for funnel plot asymmetry, $p = 0.7$)

(b) after restriction to studies unselected for age at diagnosis, family history or aggressive disease (test for funnel plot asymmetry, $p = 0.5$)

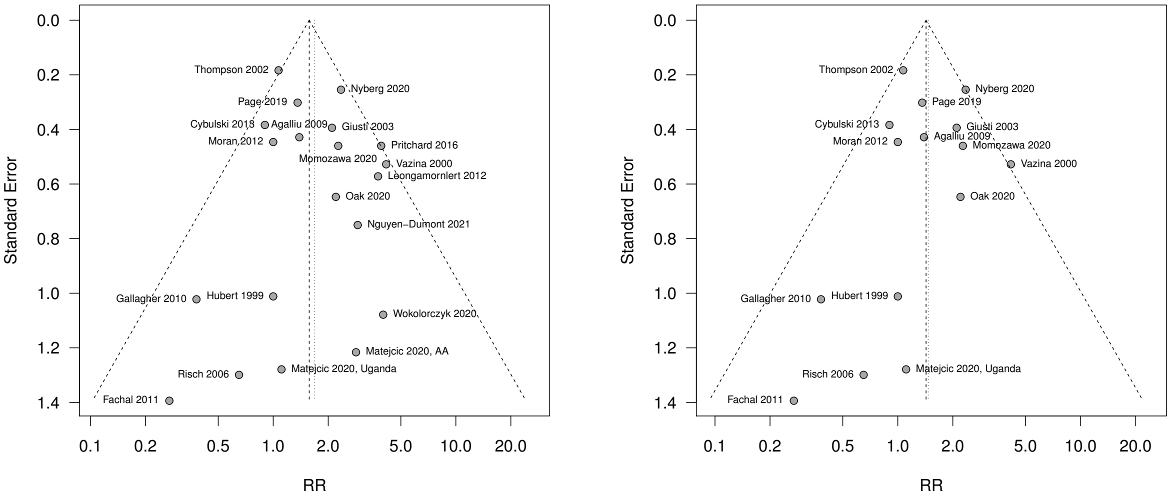

Figure S2: Funnel plots of overall *BRCA2* RR estimates.

(a) all initially considered studies (test for funnel plot asymmetry, $p = 0.5$)

(b) after restriction to studies unselected for age at diagnosis, family history or aggressive disease (test for funnel plot asymmetry, $p = 0.3$)

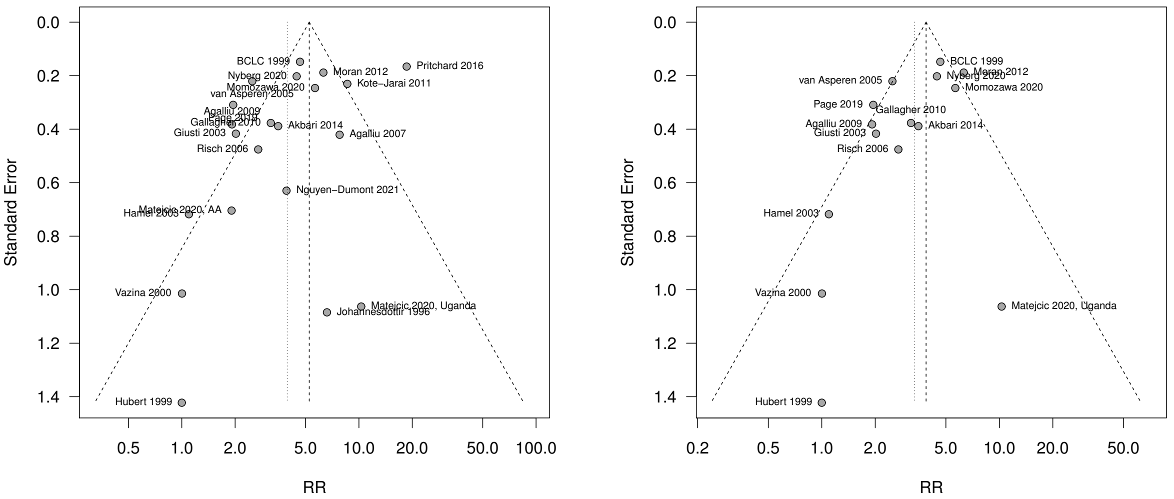

Figure S3: Forest plots of overall *BRCA1* RR estimates from studies unselected for age at diagnosis, family history or aggressive disease, restricted to studies that did not use historical controls or external population estimates.

(a) studies that did not use historical controls

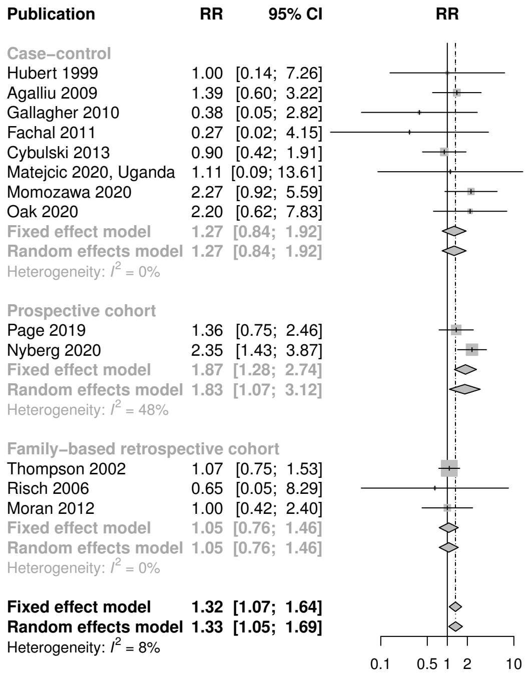

(b) studies that did not use historical controls, using the screening-adjusted estimates from the EMBRACE study

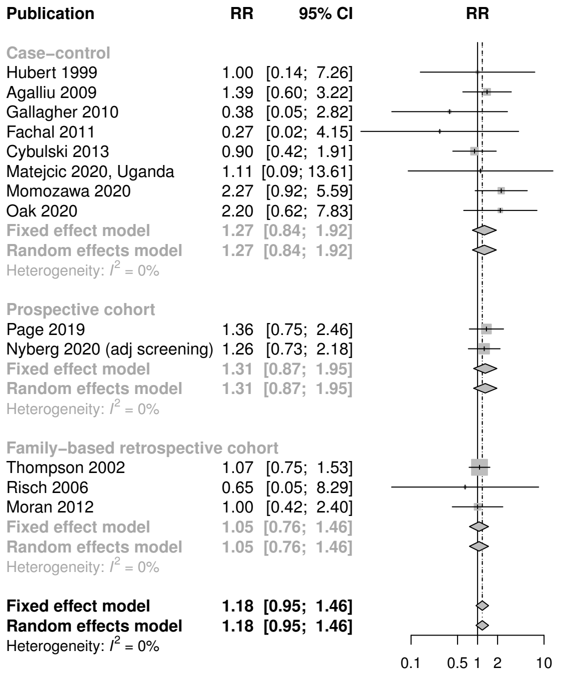

Figure S4: Funnel plots of overall *BRCA1* RR estimates from studies unselected for age at diagnosis, family history or aggressive disease, restricted to studies that did not use historical controls or external population estimates.

(a) studies that did not use historical controls (test for funnel plot asymmetry, $p = 0.5$)

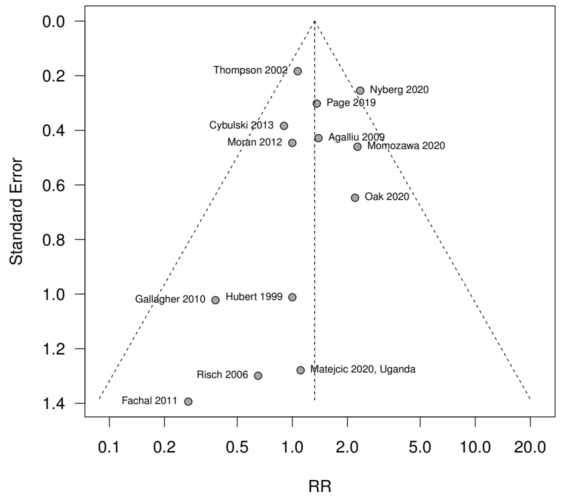

(b) studies that did not use historical controls, using the screening-adjusted estimates from the EMBRACE study (test for funnel plot asymmetry, $p = 0.4$)

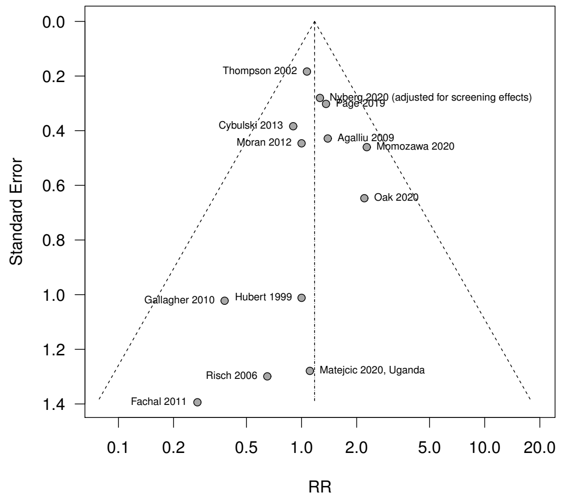

Figure S5: Forest plots of overall *BRCA2* RR estimates from studies unselected for age at diagnosis, family history or aggressive disease, by ethnicity.

(a) Ashkenazi ancestry

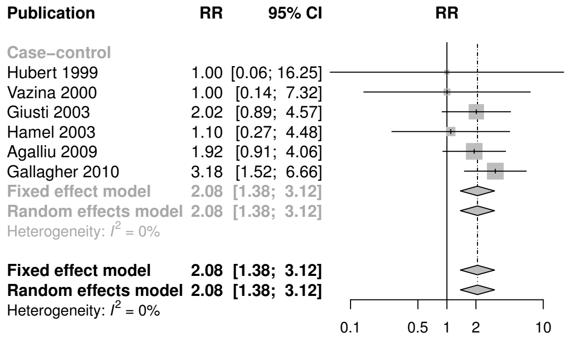

(b) non-Ashkenazi European ancestry

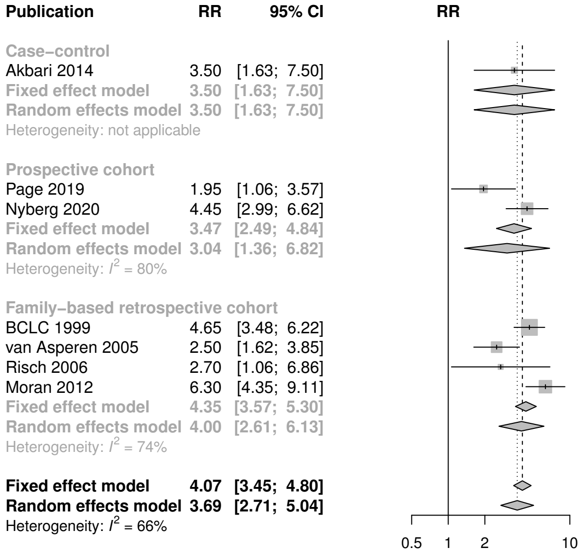

(c) non-Ashkenazi European ancestry, after exclusion of two outlier estimates

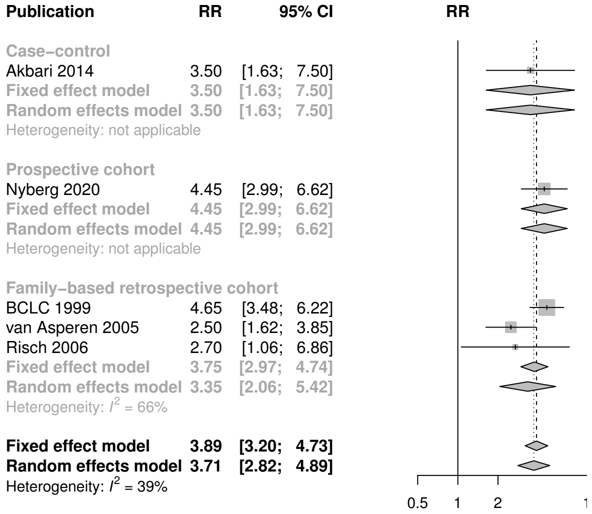

Figure S6: Funnel plots of overall *BRCA2* RR estimates from studies unselected for age at diagnosis, family history or aggressive disease, by ethnicity.

(a) Ashkenazi populations (test for funnel plot asymmetry, $p = 0.19$)

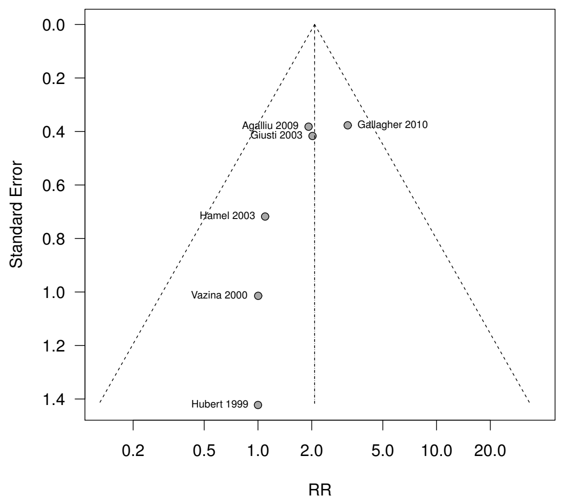

(b) non-Ashkenazi European ancestry populations (test for funnel plot asymmetry, $p = 0.099$)

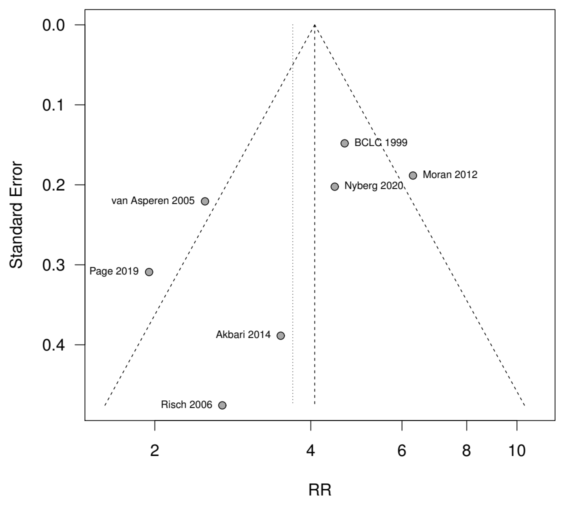

(c) non-Ashkenazi European ancestry populations, after exclusion of two outlier estimates (test for funnel plot asymmetry, $p = 0.16$)

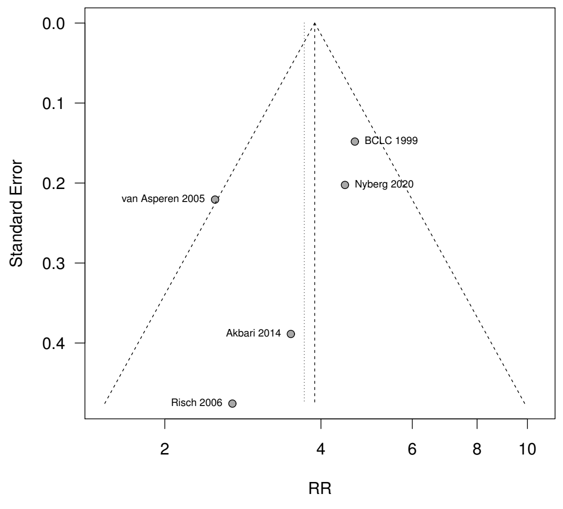

Figure S7: Forest plots of overall *BRCA2* RR estimates from studies unselected for age at diagnosis, family history or aggressive disease, by proportion of pathogenic variants located in the ovarian cancer cluster region.

(a) ≥50% PVs located in the OCCR

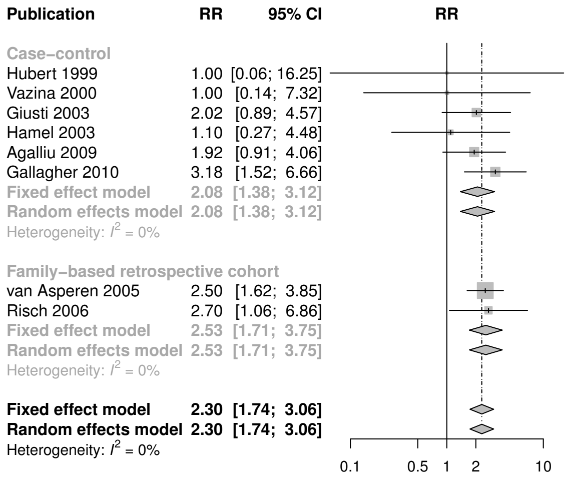

(b) <50% PVs located in the OCCR

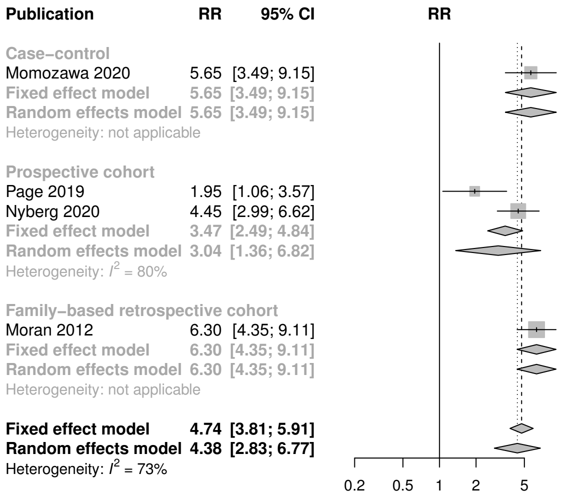

(c) Proportion of PVs located in the OCCR not determinable

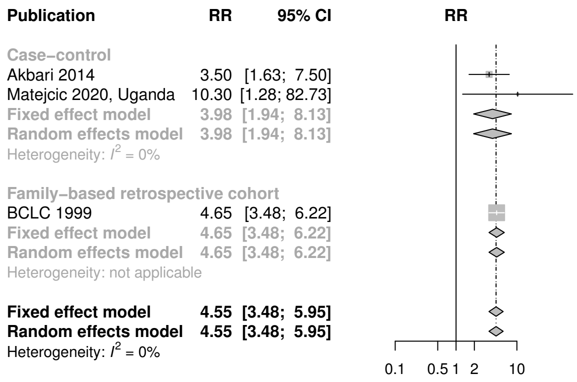

(d) <50% PVs located in the OCCR, after exclusion of one outlier estimate

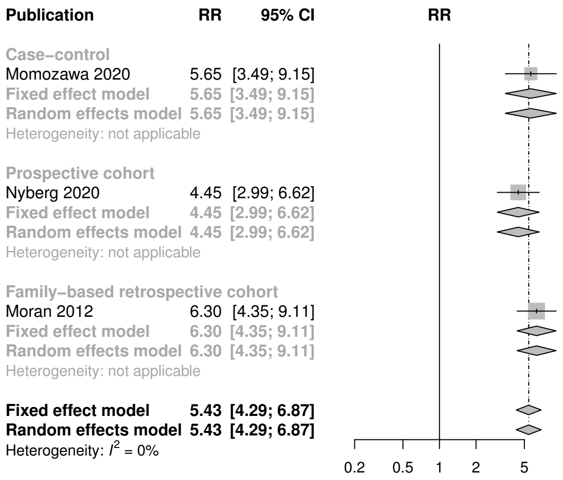

Figure S8: Funnel plots of overall *BRCA2* RR estimates from studies unselected for age at diagnosis, family history or aggressive disease, by proportion of pathogenic variants located in the ovarian cancer cluster region.

(a) ≥50% PVs located in the OCCR (test for funnel plot asymmetry, $p = 0.083$)

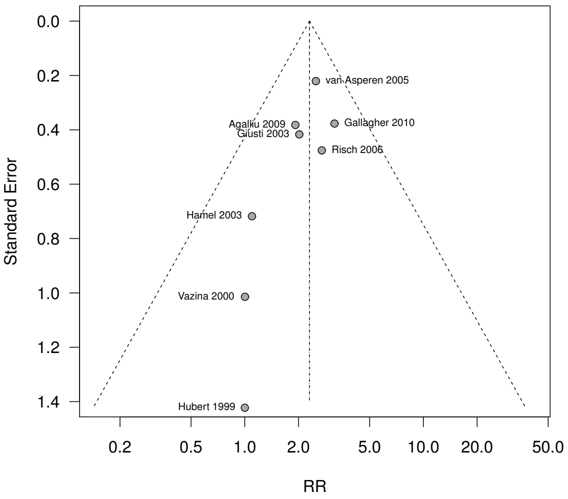

(b) <50% PVs located in the OCCR (test for funnel plot asymmetry, $p = 0.17$)

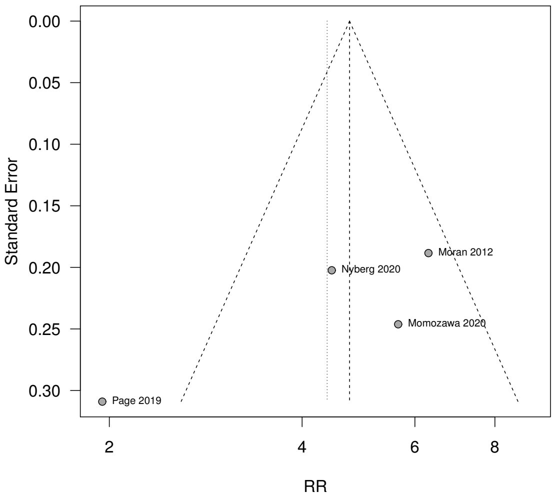

(c) Proportion of PVs located in the OCCR not determinable (test for funnel plot asymmetry, $p = 0.6$)

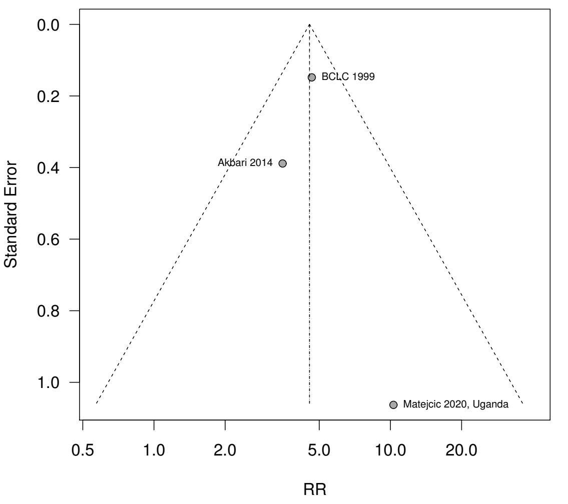

(d) <50% PVs located in the OCCR, after exclusion of one outlier estimate (test for funnel plot asymmetry, $p = 0.6$)

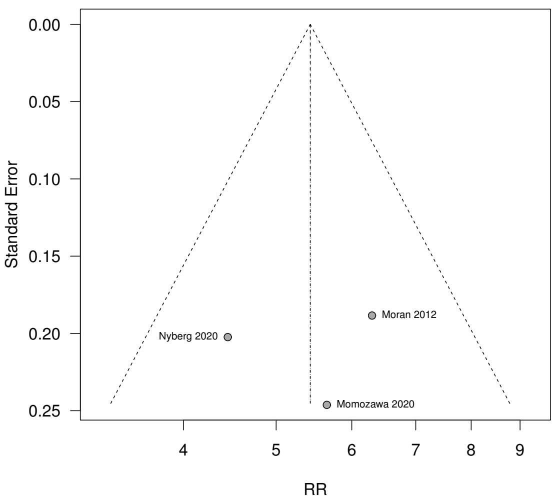

Figure S9: Meta-regression of overall *BRCA2* RR estimates from studies unselected for age at diagnosis, family history or aggressive disease, by proportion of pathogenic variants located in the ovarian cancer cluster region.

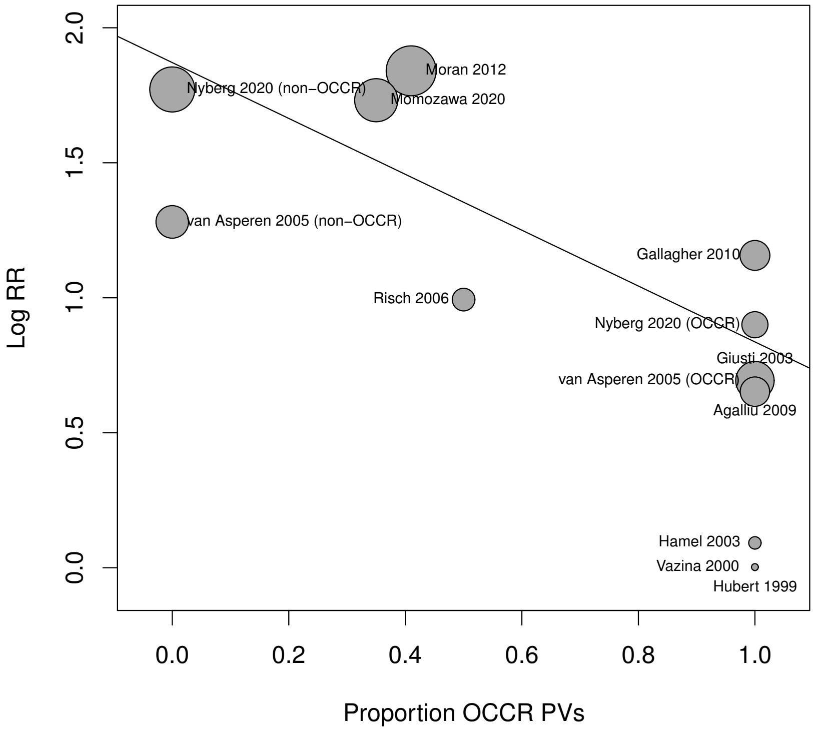

Figure S10: Forest plots of *BRCA1* RR estimates by age, by all reported age cut points.

(a) all younger age estimates, by age cut point

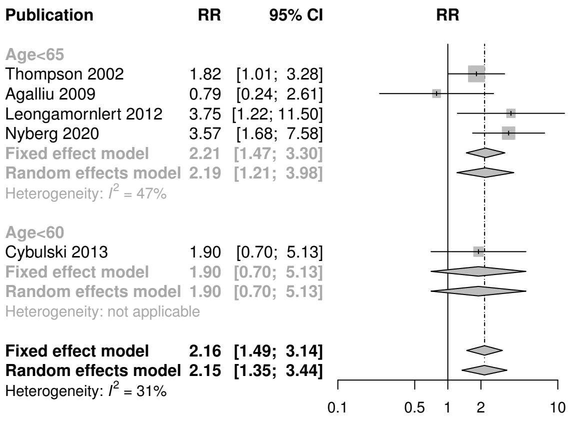

(b) all older age estimates, by age cut point

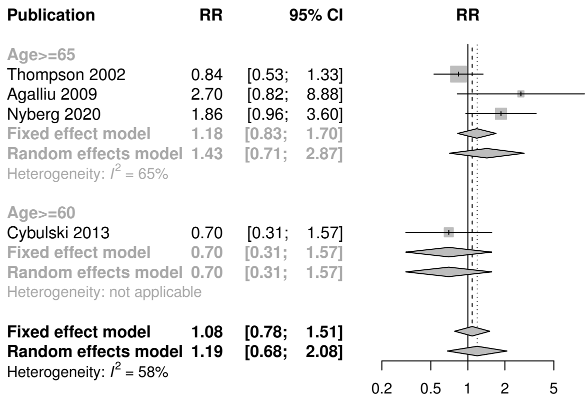

Figure S11: Forest plots of *BRCA2* RR estimates by age, by all reported age cut points.

(a) all younger age estimates, by age cut point

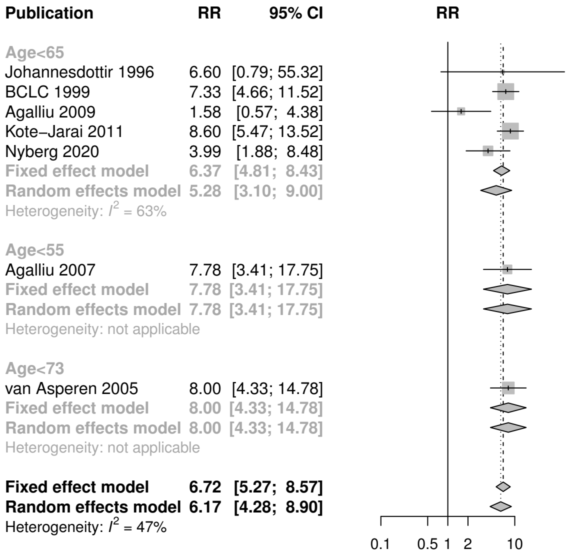

(b) all older age estimates, by age cut point

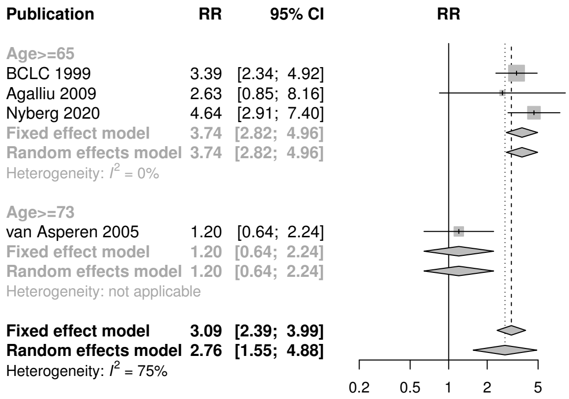

Figure S12: Forest plots of *BRCA1* RR estimates by age below or above 65 years.

(a) all age<65 years estimates

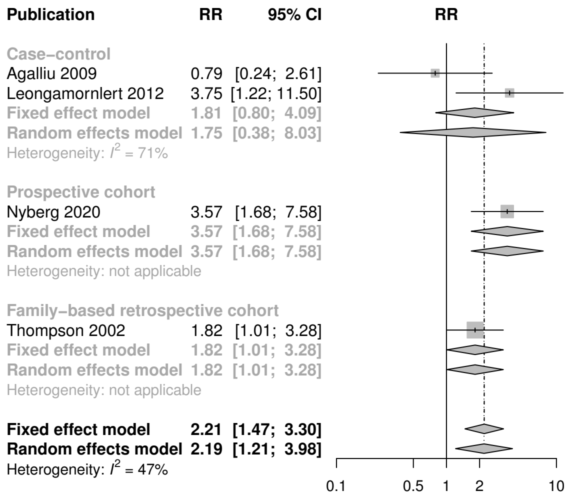

(b) all age≥65 years estimates

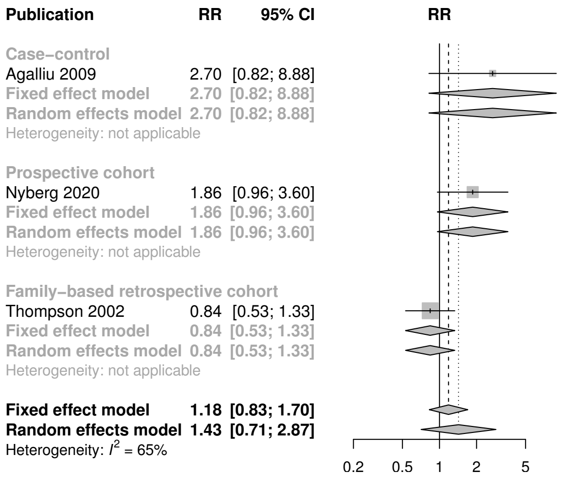

(c) age<65 years estimates, using the EMBRACE estimate adjusted for screening effects

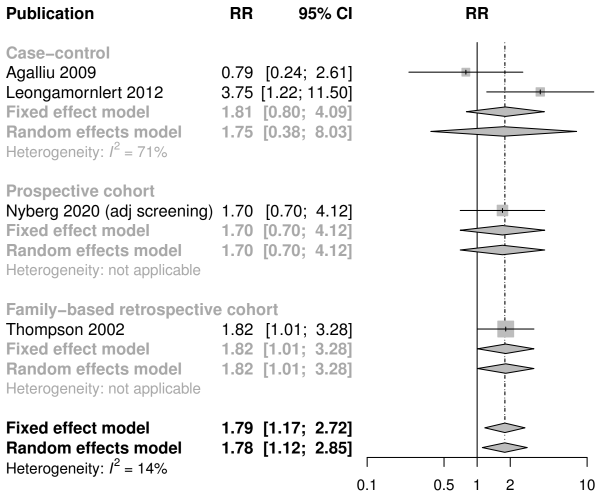

(d) age≥65 years estimates, using the EMBRACE estimate adjusted for screening effects

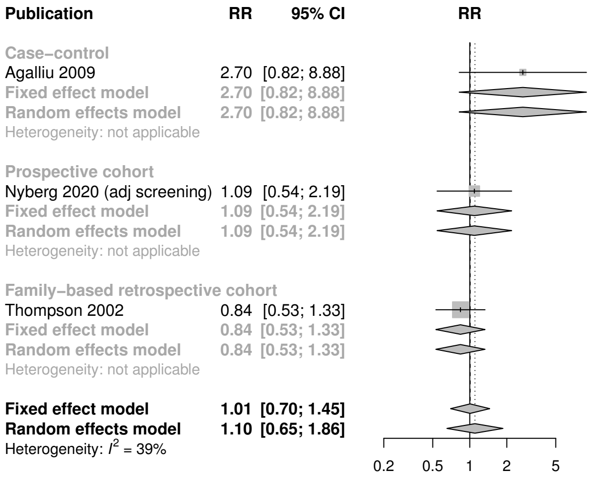

Figure S13: Funnel plots of *BRCA1* RR estimates by age below or above 65 years.

(a) all age<65 years estimates (test for funnel plot asymmetry, $p = 0.5$)

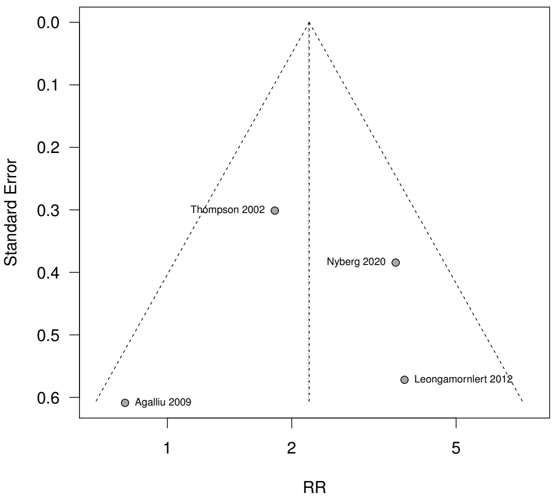

(b) all age≥65 years estimates (test for funnel plot asymmetry, $p = 0.6$)

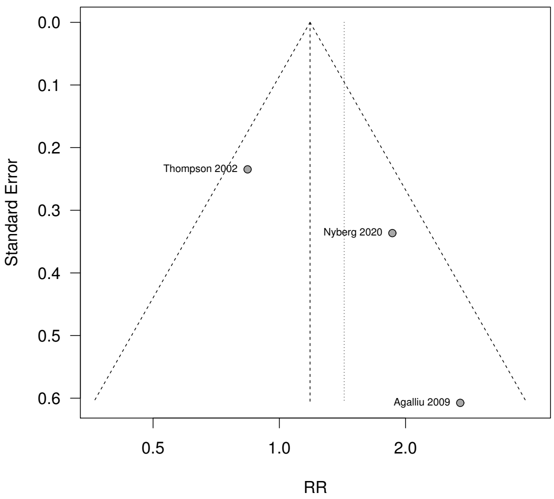

(c) age<65 years estimates, using the EMBRACE estimate adjusted for screening effects (test for funnel plot asymmetry, $p = 0.5$)

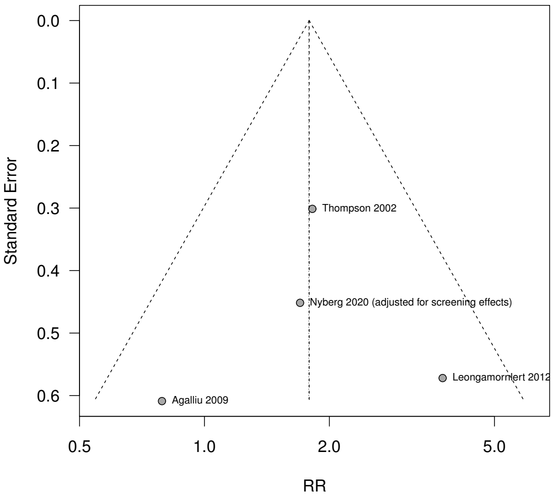

(d) age≥65 years estimates, using the EMBRACE estimate adjusted for screening effects (test for funnel plot asymmetry, $p = 0.12$)

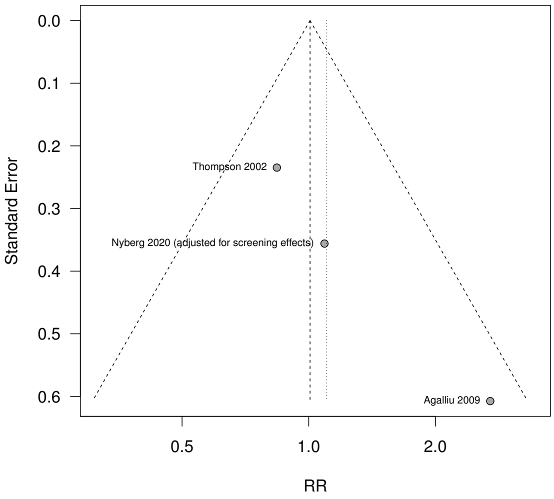

Figure S14: Forest plots of *BRCA2* RR estimates by age below or above 65 years.

(a) all age<65 years estimates

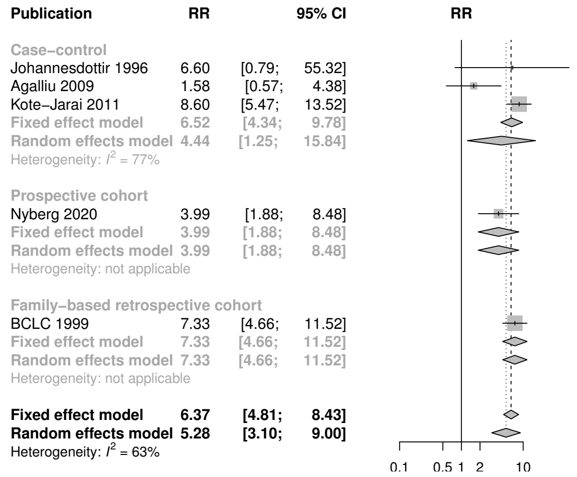

(b) all age≥65 years estimates

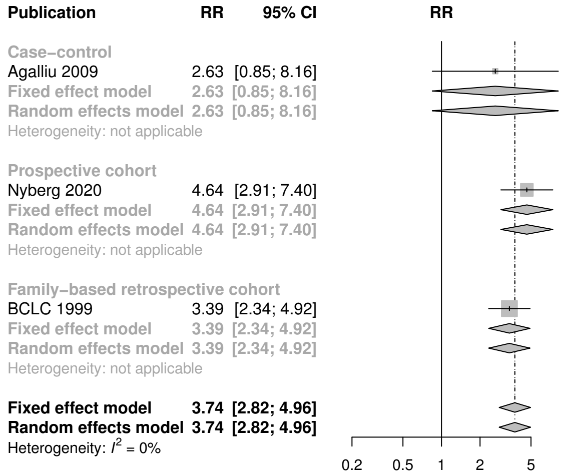

(c) age<65 years estimates for non-Ashkenazi European ancestry populations

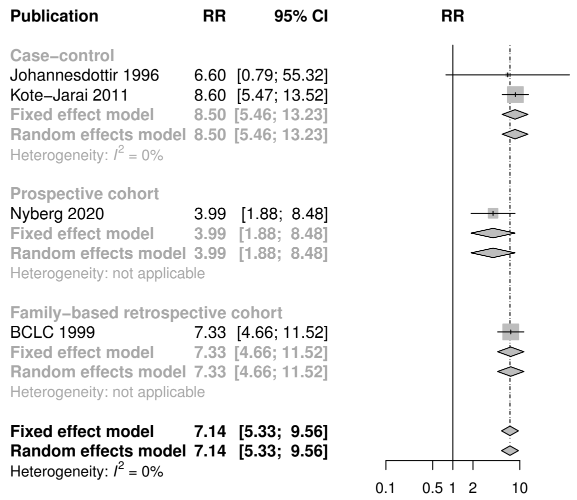

(d) age≥65 years estimates for non-Ashkenazi European ancestry populations

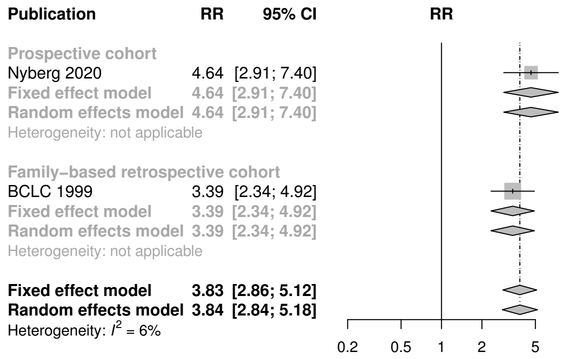

Figure S15: Funnel plots of *BRCA2* RR estimates by age below or above 65 years.

(a) all age<65 years estimates (test for funnel plot asymmetry, $p = 0.14$)

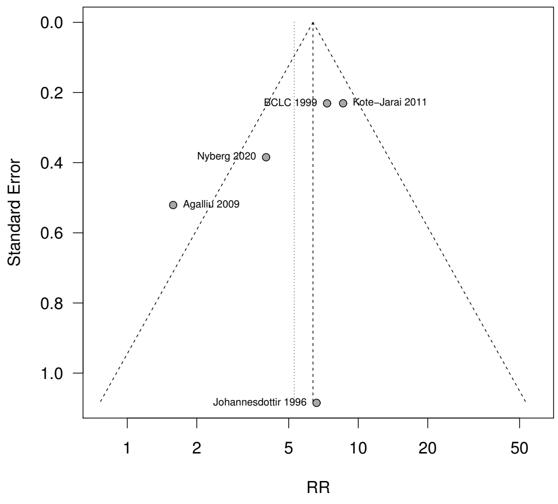

(b) all age≥65 years estimates (test for funnel plot asymmetry, $p = 0.6$)

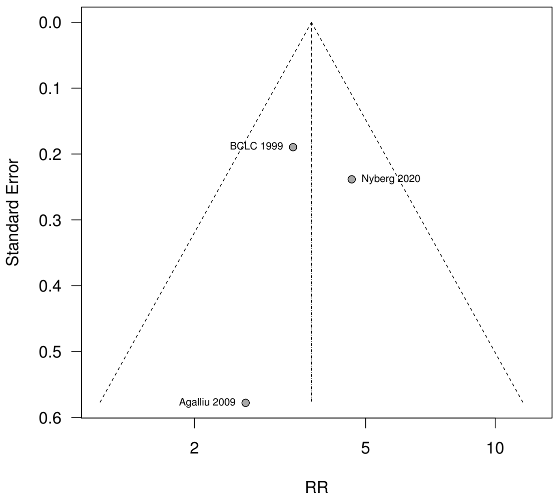

(c) age<65 years estimates for non-Ashkenazi European ancestry populations (test for funnel plot asymmetry, $p = 0.17$)

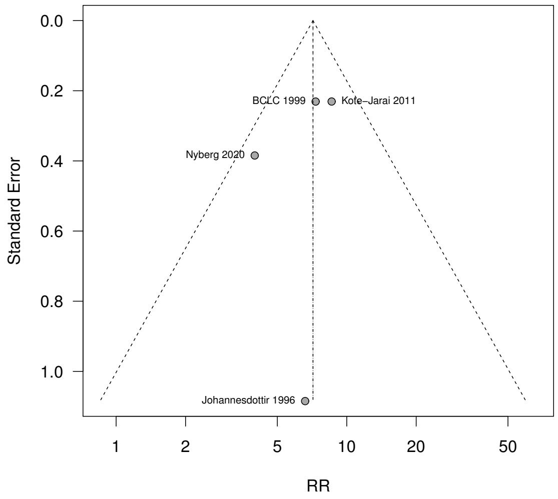

(d) age≥65 years estimates for non-Ashkenazi European ancestry populations (test for funnel plot asymmetry not done)

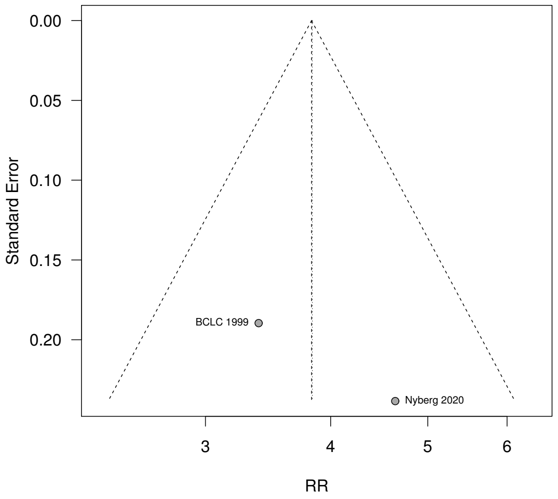

Figure S16: Forest plots of RR estimates of aggressive prostate cancer, by aggressive prostate cancer definition.

(a) *BRCA1* carriers

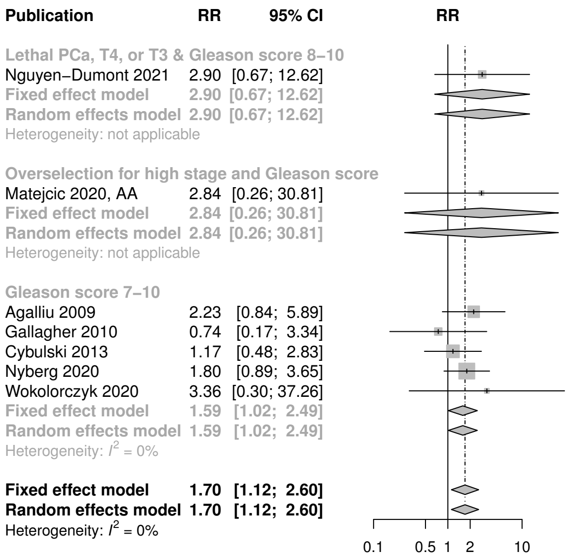

(b) *BRCA2* carriers

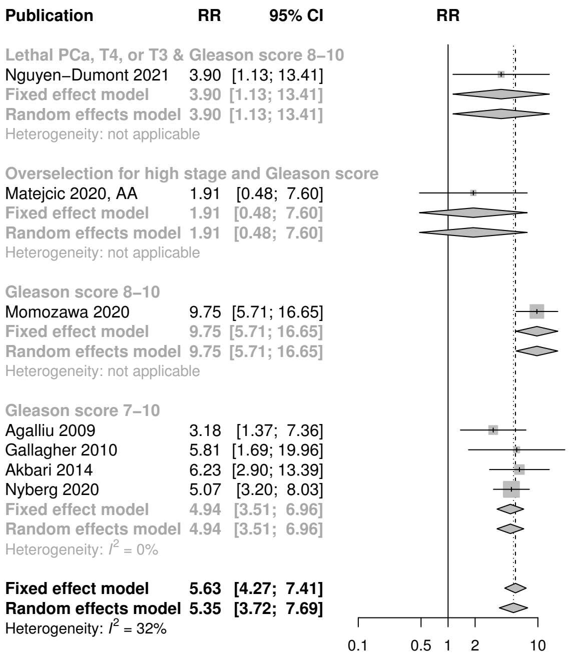

References

[1] Johannesdottir G, Gudmundsson J, Bergthorsson JT, Arason A, Agnarsson BA, Eiriksdottir G, et al. High prevalence of the 999del5 mutation in Icelandic breast and ovarian cancer patients. Cancer research. 1996;56(16):3663–3665.

[2] Breast Cancer Linkage Consortium. Cancer risks in *BRCA2* mutation carriers. Journal of the National Cancer Institute. 1999;91(15):1310–1316.

[3] Hubert A, Peretz T, Manor O, Kaduri L, Wienberg N, Lerer I, et al. The Jewish Ashkenazi founder mutations in the *BRCA1/BRCA2* genes are not found at an increased frequency in Ashkenazi patients with prostate cancer. American journal of human genetics. 1999;65(3):921–924.

[4] Vazina A, Baniel J, Yaacobi Y, Shtriker A, Engelstein D, Leibovitz I, et al. The rate of the founder Jewish mutations in *BRCA1* and *BRCA2* in prostate cancer patients in Israel. British journal of cancer. 2000;83(4):463–466.

[5] Thompson D, Easton DF, Breast Cancer Linkage Consortium. Cancer incidence in *BRCA1* mutation carriers. Journal of the National Cancer Institute. 2002;94(18):1358–1365.

[6] Giusti RM, Rutter JL, Duray PH, Freedman LS, Konichezky M, Fisher-Fischbein J, et al. A twofold increase in BRCA mutation related prostate cancer among Ashkenazi Israelis is not associated with distinctive histopathology. Journal of medical genetics. 2003;40(10):787–792.

[7] Hamel N, Kotar K, Foulkes WD. Founder mutations in BRCA1/2 are not frequent in Canadian Ashkenazi Jewish men with prostate cancer. BMC medical genetics. 2003;4(1):7.

[8] Van Asperen CJ, Brohet RM, Meijers-Heijboer EJ, Hoogerbrugge N, Verhoef S, Vasen HFA, et al. Cancer risks in BRCA2 families: estimates for sites other than breast and ovary. Journal of medical genetics. 2005;42(9):711–719.

[9] Risch HA, McLaughlin JR, Cole DEC, Rosen B, Bradley L, Fan I, et al. Population *BRCA1* and *BRCA2* mutation frequencies and cancer penetrances: a kin–cohort study in Ontario, Canada. Journal of the National Cancer Institute. 2006;98(23):1694–1706.

[10] Agalliu I, Karlins E, Kwon EM, Iwasaki LM, Diamond A, Ostrander EA, et al. Rare germline mutations in the BRCA2 gene are associated with early-onset prostate cancer. British journal of cancer. 2007;97(6):826–831.

[11] Agalliu I, Gern R, Leanza S, Burk RD. Associations of high-grade prostate cancer with *BRCA1* and *BRCA2* founder mutations. Clinical cancer research. 2009;15(3):1112–1120.

[12] Gallagher DJ, Gaudet MM, Pal P, Kirchhoff T, Balistreri L, Vora K, et al. Germline *BRCA* mutations denote a clinicopathologic subset of prostate cancer. Clinical cancer research. 2010;16(7):2115–2121.

[13] Fachal L, Gómez-Caamaño A, Celeiro-Muñoz C, Peleteiro P, Blanco A, Carballo A, et al. BRCA1 mutations do not increase prostate cancer risk: Results from a meta-analysis including new data. The prostate. 2011;71(16):1768–1779.

[14] Kote-Jarai Z, Leongamornlert D, Saunders E, Tymrakiewicz M, Castro E, Mahmud N, et al. *BRCA2* is a moderate penetrance gene contributing to young-onset prostate cancer: implications for genetic testing in prostate cancer patients. British journal of cancer. 2011;105(8):1230–1234.

[15] Leongamornlert D, Mahmud N, Tymrakiewicz M, Saunders E, Dadaev T, Castro E, et al. Germline *BRCA1* mutations increase prostate cancer risk. British journal of cancer. 2012;106(10):1697–1701.

[16] Moran A, O'Hara C, Khan S, Shack L, Woodward E, Maher ER, et al. Risk of cancer other than breast or ovarian in individuals with *BRCA1* and *BRCA2* mutations. Familial cancer. 2012;11(2):235–242.

[17] Cybulski C, Wokołorczyk D, Kluźniak W, Jakubowska A, Górski B, Gronwald J, et al. An inherited NBN mutation is associated with poor prognosis prostate cancer. British journal of cancer. 2013;108(2):461–468.

[18] Akbari MR, Wallis CJD, Toi A, Trachtenberg J, Sun P, Narod SA, et al. The impact of a BRCA2 mutation on mortality from screen-detected prostate cancer. British journal of cancer. 2014;111(6):1238–1240.

[19] Pritchard CC, Mateo J, Walsh MF, De Sarkar N, Abida W, Beltran H, et al. Inherited DNA-repair gene mutations in men with metastatic prostate cancer. New England journal of medicine. 2016;375:443–453.

[20] Page EC, Bancroft EK, Brook MN, Assel M, Al Battat MH, Thomas S, et al. Interim results from the IMPACT study: evidence for prostate-specific antigen screening in BRCA2 mutation carriers. European urology. 2019;76(6):831–842.

[21] Matejcic M, Patel Y, Lilyquist J, Hu C, Lee KY, Gnanaolivu RD, et al. Pathogenic variants in cancer predisposition genes and prostate cancer risk in men of African ancestry. JCO precision oncology. 2020;4:32–43.

[22] Momozawa Y, Iwasaki Y, Hirata M, Liu X, Kamatani Y, Takahashi A, et al. Germline Pathogenic Variants in 7636 Japanese Patients With Prostate Cancer and 12 366 Controls. JNCI: Journal of the National Cancer Institute. 2020;112(4).

[23] Nyberg T, Frost D, Barrowdale D, Evans DG, Bancroft E, Adlard J, et al. Prostate cancer risks for male *BRCA1* and *BRCA2* mutation carriers: a prospective cohort study. European urology. 2020;77(1):24–35.

[24] Oak N, Cherniack AD, Mashl RJ, Hirsch FR, Ding L, Beroukhim R, et al. Ancestry-specific predisposing germline variants in cancer. Genome medicine. 2020;12:51.

[25] Wokołorczyk D, Kluźniak W, Huzarski T, Gronwald J, Szymiczek A, Rusak B, et al. Mutations in ATM, NBN and BRCA2 predispose to aggressive prostate cancer in Poland. International journal of cancer. 2020;147(10):2793–2800.

[26] Nguyen-Dumont T, Dowty JG, MacInnis RJ, Steen JA, Riaz M, Dugué PA, et al. Rare germline pathogenic variants identified by multigene panel testing and the risk of aggressive prostate cancer. Cancers. 2021;13(7):1495.
